# Implementing proximity care for people with multiple sclerosis in Italy: the bottom-up approach of the StayHome project

**DOI:** 10.1007/s00415-024-12749-8

**Published:** 2025-01-07

**Authors:** M. Filippi, P. Gallo, C. Gasperini, G. A. Marfia, C. Avolio, R. Bergamaschi, M. Capobianco, M. Dotta, L. Grimaldi, G. Lus, F. Patti, E. Pucci, R. Quatrale, P. Solla, P. Bandiera, C. Angioletti, M. C. Gallottini, S. Parretti, L. Pinto, F. Pavone, S. Sanzone, the StayHome Project Working Group

**Affiliations:** 1 Neurology Unit and Multiple Sclerosis Center, Neurorehabilitation Unit, and Neurophysiology Service, IRCCS San Raffaele Scientific Institute, Via Olgettina, 60, 20132 Milan, Italy; 2 Neuroimaging Research Unit, Division of Neuroscience, IRCCS San Raffaele Scientific Institute, Milan, Italy; 3 Vita-Salute San Raffaele University, Milan, Italy; 4 UOS Day Hospital, Telemedicine and Centre for Advanced Therapies in Neurology, Department for Neurosciences, DNS, University Hospital, Padua, Italy; 5 Multiple Sclerosis Centre of the Veneto Region, Padua, Italy; 6 Neurological Division, S. Camillo Forlanini Hospital, Rome, Italy; 7 Multiple Sclerosis Clinical and Research Unit, Fondazione Policlinico Tor Vergata, Department of Systems Medicine, Tor Vergata University, Rome, Italy; 8 Department of Medical and Surgical Sciences, University of Foggia, Foggia, Italy; 9 Multiple Sclerosis Center, Policlinico Hospital, Foggia, Italy; 10 Multiple Sclerosis Research Center, IRCCS Mondino Foundation, Pavia, Italy; 11 SC Neurologia, ASO S. Croce e Carle, Cuneo, Italy; 12 Multiple Sclerosis Center, Michele and Pietro Ferrero Hospital, Verduno, Cuneo, Italy; 13 UOC Neurologia, Fondazione Istituto G. Giglio, Contrada Pietrapollastra Snc, Cefalù, Palermo, Italy; 14 Multiple Sclerosis Center, II Division of Neurology, University of Campania “L. Vanvitelli”, C.I.R.N. (Centro Interuniversitario per le Ricerche in Neuroscienze), Naples, Italy; 15 UOS Sclerosi Multipla, Gaspare Rodolico Hospital, Catania, Italy; 16 UOC Neurological Unit, AST Fermo, Fermo, Italy; 17 Department of Neurological Sciences, Neurology Unit, Ospedale dell’Angelo, Venice, Italy; 18 Neurological Clinic AOU Sassari, Department of Medicine, Surgery e Pharmacy, University of Sassari, Sassari, Italy; 19 Italian Multiple Sclerosis Society (AISM), Genoa, Italy; 20 IQVIA, Milan, Italy; 21 Biogen, Milan, Italy

**Keywords:** Proximity care, Multiple sclerosis care pathway, Organizational improvement

## Abstract

**Objective:**

In Italy, around 137,000 people live with multiple sclerosis, facing organizational complexities due to the current model’s limited focus on proximity care. This project aims to define a proximity model, in accordance with recent developments in the Italian healthcare landscape, engaging over 150 healthcare stakeholders and potentially impacting approximately 14,000 patients.

**Methods:**

An analysis was pursued to map the multiple sclerosis pathway, followed by interviews to capture the actual implementation in Italian Multiple Sclerosis Centers. Through the experts’ insights, an optimal proximity care pathway and a Maturity Model framework were defined. This model was piloted in 14 centers, and a preliminary pre-post analysis was performed to evaluate initial improvements. Finally, a two-round Delphi method validated the Maturity Model dimensions and a set of key performance indicators. A scientific board including neurologists, patient associations and scientific associations, supervised project progresses and methodologies.

**Results:**

The Pilot study results show an overall increase in the centers’ positioning within the Maturity Model levels after adopting center-specific action plans. To generalize the model, the Delphi panel validated a subset of process, volume, outcome and patient experience indicators (9 of 26 proposed) along with qualitative dimensions defining the Maturity Model (13 of 20 proposed), therefore, outlining a comprehensive monitoring framework for the multiple sclerosis patient pathway.

**Conclusion:**

This study shows, for the first time in Italy, the efficacy of a bottom-up approach in addressing organizational challenges within the current multiple sclerosis scenario. This integrated model offers future opportunity for replication across various care pathways and settings.

**Supplementary Information:**

The online version contains supplementary material available at 10.1007/s00415-024-12749-8.

## Introduction

In 2023, Italy counted 137,000 people with multiple sclerosis (pwMS), one of the most common and relevant chronic neurological diseases, with a prevalence of 0.22% [1]. MS is unpredictable and heterogeneous among patients, and affects all the functions of the central nervous system, leading to severe symptoms and irreversible disabilities that require continuous care, therefore calling for a multidisciplinary approach [2–4]. The disease has a typical onset in young adults at approximately 30 years of age, however, thanks to therapies that can control and slow down the progression of the pathology, severe levels of disability are deferred up to 25 years after the diagnosis [1]. Although there has been considerable progress in the treatment options available to pwMS, organizational aspects still leave room for improvement. It has been pointed out that in Italy 50% of pwMS do not receive the care they should, due to the long waiting lists for health services, also impacted by the effects of the Coronavirus pandemic, determining delays and changes in the care pathway [1].

Today MS care, as for most health conditions in Italy, is centered around hospitals and more specifically in Multiple Sclerosis Centers (MSC). Italy counts approximately 240 MSCs, which are typically affiliated with neurological departments in hospitals, and staffed with specialized personnel to ensure appropriate care [5]. Accordingly, 90% of pwMS consider MSCs the reference point for the health service delivery, and rarely utilize territorial facilities throughout the care pathway. This is also due to the fact that disease modifying therapies (DMTs), can be prescribed exclusively by authorized centers, typically MSCs [6]. To this regard, they are the crucial hub of MS management, however, different activities within the care pathway could be delivered in different settings, therefore, it is relevant to establish an MS network to ensure continuity of care. An initial shift has already begun in this realm, supported by the new health directives and funds allocated for healthcare transformation following Covid-19 [7, 8], with the aim of enhancing territorial assistance and digitalization. Accordingly, MSCs are becoming coordinators of care, defining the care pathway beyond the hospital setting, including territorial facilities. While upholding their central role, MSCs should be seamlessly integrated into the territorial network to guarantee access to care for all pwMS and ensure continuity of care. This integration enables the monitoring of pwMS and early detection of any hidden symptoms, thereby preventing complications. This would help reduce understaffing and organizational complexity.

In this scenario, in 2021 AGENAS (the national agency for regional health services) has institutionalized a national integrated care pathway (ICP) underlining the new direction taken by the healthcare system to be adapted to MS [2]. The objective of the ICP is to establish guidelines to enforce the right of pwMS to have access to homogeneous diagnostic and therapeutic services on the national territory. The National Health System (Servizio Sanitario Nazionale, SSN) is highly decentralized, where each region defines a different organizational model for delivering health services. While this governance model aims to enhance efficiency and management effectiveness, it results in significant heterogeneity among regions [9]. Therefore, the ICP serves as general guideline to be adopted and localized to the regional context. Assuring continuity of care collaboration between MSCs and the territory is essential, together with the optimization of human, structural and economic resources [1]. In addition to defining the patient pathway, the AGENAS document promotes the process of continuous improvement through the monitoring of key performance indicators (KPI) [2].

Currently, there is no comprehensive model that delineates the practical implications of the evolving paradigm of MS management, therefore, assessing this gap is relevant to enhance pwMS experience, increase quality of life, and reduce use of health services due to complications. The aim of the StayHome project is to overcome the limitations of the current models of care by defining a new model of proximity care through a bottom-up approach, in line with the new national guidelines. This will be achieved by defining a practical framework outlined in a Maturity Model and KPIs.

## Methods

### Study design and participants

The StayHome project was initiated in 2021 with Biogen’s support, and IQVIA as operational partner to manage the project’s methodological approach and execution. IQVIA Italy’s Implementation Science Team (hereinafter operative team) led the development of the project’s phases, drawing on their specialized expertise in optimizing clinical pathways and healthcare processes.

The StayHome project has been conceived as a multistep study that aims to define an optimal proximity care model for managing MS. In its preliminary phase, a steering committee comprising neurologists, representatives of the Italian Society of Neurology (Società Italiana di Neurologia, SIN), and the Italian Multiple Sclerosis Society (Associazione Italiana Sclerosi Multipla, AISM) was established to provide oversight and validation throughout the different project activities, framework development, and methodological deployment. AISM’s participation in the steering committee ensured that the patient perspective was consistently represented at every stage of the project, with a focus on addressing needs and experiences.

**Fig. 1:**
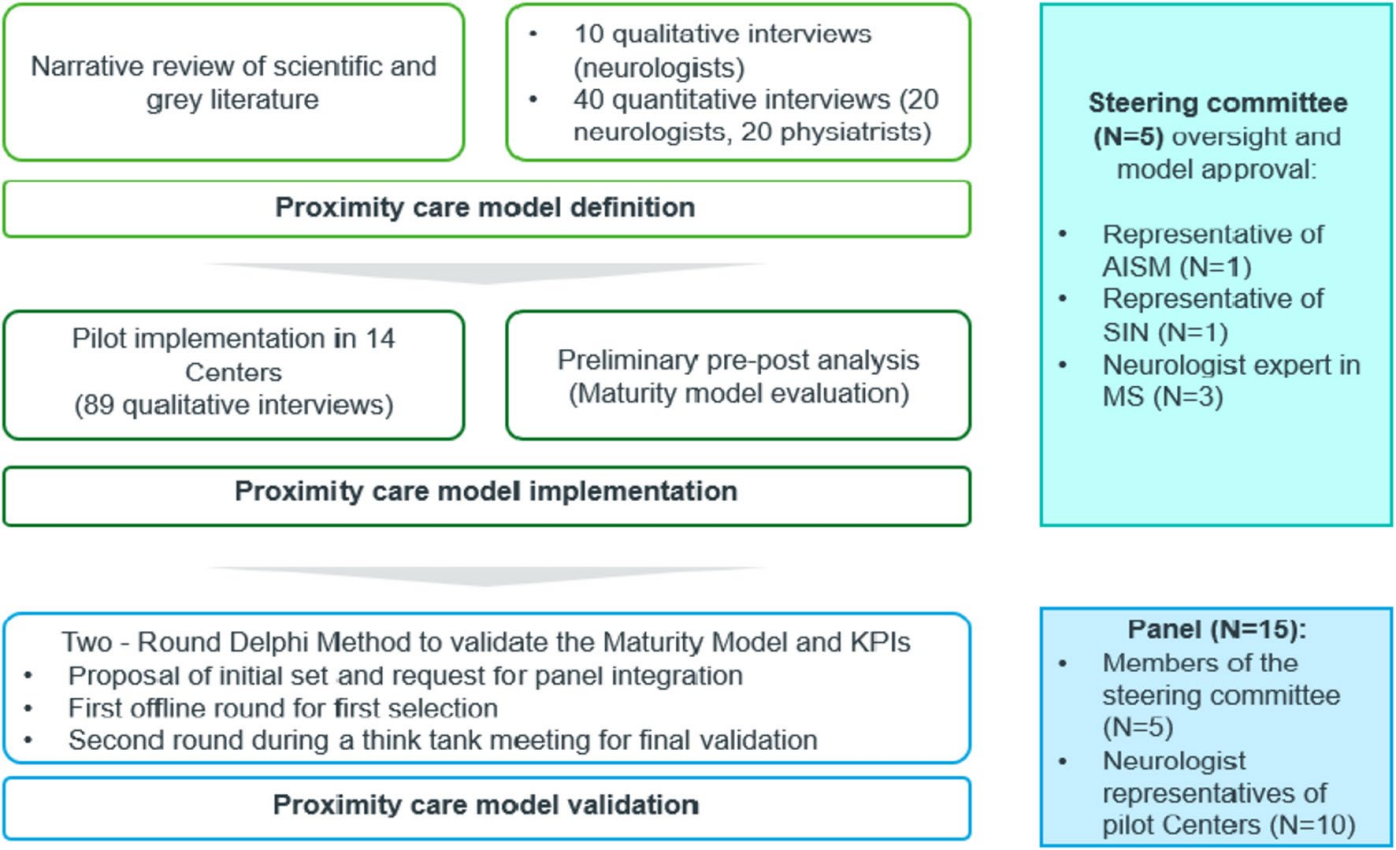
Representation of the methodological steps from the model definition to its validation

The Standards for Quality Improvement Reporting Excellence (SQUIRE) guidelines were adopted to provide a framework for reporting new knowledge about how to improve healthcare [10]. The steps from the model definition to its validation are reported in the following paragraphs and summarized in Fig. 1.

### MS proximity model definition

First, a narrative review of scientific and grey literature was conducted to outline the current MS care pathway and collect experiences of proximity models. Good practices, guidelines, care pathways and studies describing or experimenting with MS proximity care management, in national and international contexts, were collected using scientific and generic search engines (Pubmed, Google Scholar). In order to summarize the findings and represent how, in theory, MS should be managed, the care delivery value chain methodology was adopted. This methodology is rooted in value-based healthcare principles and enables thorough analysis and enhancement of socio-healthcare and therapeutic processes for specific medical conditions. Unlike traditional approaches, which focus on individual procedures or healthcare settings, this methodology considers the entire patient journey from initial diagnostic suspicion to follow-up, ensuring a holistic and patient-centered perspective [11–14].

Once the model was outlined, a mixed-methods approach was adopted. Accordingly, qualitative semi-structured interviews with 10 neurologists were conducted alongside with a quantitative survey administered to 40 MS experts, inclusive of 20 neurologists and 20 physiatrists. These figures have been selected for their role in the patient pathway, the neurologist being the owner of the whole MS clinical care journey and the physiatrist being pivotal in the rehabilitation phase. Qualitative interviews investigated all the phases of the MS patient journey, being: identification of condition suspect, diagnosis, treatment, clinical follow-up and rehabilitation pathway for different levels of disability, assessed with the Expanded Disability Status Scale (EDSS). The semi-structured interviews were conducted by two members of the operative team who attended each interview simultaneously both taking notes independently. After all the interviews were performed, both researchers reviewed and analyzed the information, and identified key themes and recommendations related to the MS patient journey. This approach allowed for cross-validation of findings and minimized potential biases. The thematic analysis enabled the exploration of recurring patterns and emerging insights that either support or challenge the current pathway defined in literature.

The same topics were addressed in the survey administered through a Computer Assisted Web Interviewing (CAWI) questionnaire reported in Supplementary 1. It was designed to gather quantitative data to complement and validate the qualitative insights obtained from the interview. The results from the survey were analyzed using various descriptive statistical methods to gain a comprehensive understanding of the quantitative aspects of MS management. This involved evaluating the volumes, frequencies, distributions, as well as the staffing levels and specialized dedicated pathways within the scope of MS management. The integration of qualitative and quantitative methods facilitated a comprehensive evaluation of the current clinical pathway for MS, along with insights into how experts believe patients should be optimally managed. Synthesizing insights derived from the scoping analysis and data collected from qualitative and quantitative interviews, the final optimal MS care management model was designed, including the guiding pillars of proximity care, the optimal clinical pathway, and a monitoring system. The latter included quantitative KPIs drawn upon the AGENAS MS ICP [2] and a qualitative Maturity Model framework. The Maturity Model was designed in order to comprehensively evaluate various phases of the pathway, including initial diagnostic suspicion, diagnosis, therapy, care enrollment, neurological follow-ups, and rehabilitation pathways. For each phase of the framework, different dimensions capturing essential elements of the care pathway were identified, with four levels of maturity for each dimension, where the fourth level represented the optimal care pathway. The finalized model was approved by the steering committee.

### Pilot implementation

#### Care pathway analysis

After being approved by the steering committee, the optimal care model was tested on Italian MSCs. 14 hub MSCs were selected, out of the 240 centers specialized in MS care in Italy (Table 1). Eligibility criteria were formulated to address the considerable heterogeneity inherent in the Italian healthcare system. Accordingly, geographical distribution of MSCs was deemed essential to ensure a representative sample reflecting the Italian healthcare landscape. Furthermore, the selection process prioritized diversity in the capacities of chosen centers to manage pwMS, thereby enabling the analysis of MS facilities of varying sizes. This approach aimed at enhancing generalizability of the study to the national context. Following this selection process, invitations were extended to the centers to participate in the project and their involvement was entirely voluntary.

**Table 1:** Characteristics of the 14 MSCs

MSC	Location	Volume of patients*	% of patient from the province	% of patients from the region*	% of patients out of region	N. of neurologists	N. of case managers	N. of nurses
A	South	1100	95	5	0	5	1	1
B	South	2000	80	19	1	6	2	2
C	Center	1900	85	10	5	5	0	9
D	Center	2000	60	30	10	5	1	3
E	North	400	80	16	4	2	0	3
F	South	1500	30	55	15	4	1	3
G	Center	1000	90	9	1	3	0	NA
H	North	1100	50	25	25	6	1	NA
I	South	1100	80	18	2	4	1	2
J	North	300	90	10	0	3	0	2
K	Center	440	65	30	5	3	0	NA
L	North	3500	50	20	30	5	0	6
M	Nord	300	93	7	0	4	1	NA
N	North	2000	55	45	5	4	0	5

14 MSCs were included in the pilot phase, distributed on the national territory to gain a representative sample of the Italian MS care

*Data are relative to the moment of evaluation, therefore currently there could be fluctuations in volume of patients due changes in incidence of the disease; % of patients from the region refers to patients that are not residents in the province of the MSC but in the same region

NA: information not available

The pilot project involved an initial clinical pathway analysis [15, 16]. This methodological approach entails modeling a care pathway within a healthcare system, aiming to visually represent the clinical journey through system diagrams, flowcharts or maps, capturing the logical sequence characterizing the diagnosis, treatment and rehabilitation processes for the specific health condition [16]. In order to map the MSCs care pathways, a total of 89 semi-structured interviews were conducted across the 14 facilities. Six of these centers had participated in the initial phase of the MS proximity model definition, however, different neurologists were interviewed in this second phase, therefore, no professional was interviewed twice. The number of interviewees per center ranged from 4 to 11, depending on the center’s size and the availability of professionals. Interviewees included a large array of professionals, such as, neurologists, nurses, case managers, physiatrists, physiotherapists, psychologists, data managers, home care multidisciplinary teams, hospital pharmacists, medical coordinators, neuroradiologists and administrative personnel. Interviews focused on analyzing organizational aspects related to the health service delivery in the phases of the MS care pathway, thereby identifying the specificities of each MSC’s organizational model. As for the previous phase, two members of the operative team attended each interview simultaneously, both independently taking notes. Following the completion of all interviews, both researchers reviewed and analyzed the information, and subsequently outlined the patient’s organizational flow and specificities in MS management in a report. Once the processes were outlined, care pathways were compared to the optimal MS model defined in the previous phase, to identify improvement areas. In order to have a comprehensive overview of the MSC’s baseline, each center was assessed using four organizational KPIs, the first four KPIs are reported in Supplementary 2, and the Maturity Model framework. To do so, centers were asked to fill in an online self-assessment form, reported in Supplementary 3, where they had to select one of the four levels for each dimension of the model. The levels were not ordered and MSCs could select the description of the levels which were presented in random order. By combining the KPI data and the Maturity Model positioning, a detailed picture of each center’s MS care management emerged. Critical areas were identified based on lower levels of the Maturity Model and suboptimal performance in KPIs. This analysis helped pinpoint specific gaps between the current baseline and the optimal proximity model. Practical solutions were proposed aimed at enhancing the four pillars of the model, addressing both the identified KPI shortcomings and the Maturity Model dimensions. After discussion and refinement with each individual center, these solutions were formalized into a detailed action plan providing guidance on strategies for improvement.

#### Preliminary pre-post analysis

A pre-post preliminary observational analysis was performed to assess the centers’ organizational improvement following the definition of an action plan. This evaluation involved capturing qualitative insights through the progression on the Maturity Model. Accordingly, centers were repositioned on the Maturity Model framework after at least 6 months from the first assessment, through the compilation of the self-assessment form. Three MSCs were not included in the pre-post analysis because the minimum period of 6 months defined had not yet passed. The difference between the two assessment periods was then calculated for each center to measure the preliminary impact of the action plan.

### Model validation

To ensure the validity and reliability of the defined model, a two-round Delphi analysis was conducted. This method engaged experts in the field, to provide a consensus-based validation of the KPIs and the Maturity Model, therefore providing comprehensive validation of the assessment tools and methodologies employed in the project.

An initial phase of document analysis was performed to select outcomes, processes, volume and patient experience KPIs. The analysis focused on both national and local Italian ICPs [17–27] in addition to an international literature analysis. A set of 25 KPIs was identified for the first round, including the KPIs measured in the pilot phase. Additionally, qualitative dimensions to assess the organizational model of MSCs were considered. The initial pool of 19 dimensions was identified drawing upon the Maturity Model employed in the MSCs assessment in the pilot phase.

The initial set of KPIs and dimensions were presented to a panel of 15 participants, who contributed by suggesting additional elements for inclusion in the preliminary pool. The panelists, including neurologists from the MS centers involved in the pilot, representatives of SIN, and AISM, voluntarily participated. Once the preliminary set of KPIs and dimensions were identified, the first round of the Delphi method started. Each panelist completed an online form, rating each KPI based on four criteria: general relevance, scientific evidence, measurability, and actionability. Dimensions were assessed only on their general relevance. Ratings were assigned using a 5-point Likert scale, ranging from 1 being very low, to 5 being very high. Descriptive statistics were then calculated for each question, after the completion of the first round.

Selection criteria for KPIs were the following: if 75% or more of the responses rated the KPI as 4 or higher on the Likert scale for at least 3 out of the 4 criteria, the indicator was deemed eligible for inclusion. Conversely, if 75% or more of the responses rated the KPI as lower than 2 on the Likert scale for at least 3 out of the 4 criteria, the indicator was excluded. Any KPI that did not meet these criteria was categorized as uncertain and subject to further evaluation in the second round. Similarly, for dimensions, the selection criteria were as follows: if 75% or more of the responses rated a dimension as 4 or higher on the Likert scale for the general relevance criterion, the indicator was considered eligible for inclusion. Conversely, if 75% or more of the responses rated a dimension as lower than 2 on the Likert scale, the indicator was excluded. Dimensions that did not meet these criteria were also deemed uncertain and underwent further assessment in the second round.

Results of the first phase were shared with the panel during a think tank meeting, showing the included KPIs and dimensions, along with the percentage of rates higher than or equal to 4, for the included elements. During the meeting, the second round was performed to vote on uncertain elements. Panelists completed an anonymous online form, voting for the inclusion or exclusion of each element. Inclusion required at least 2/3 of votes, with elements failing to meet this threshold being excluded.

## Results

### Optimal care pathway definition

**Fig. 2:**
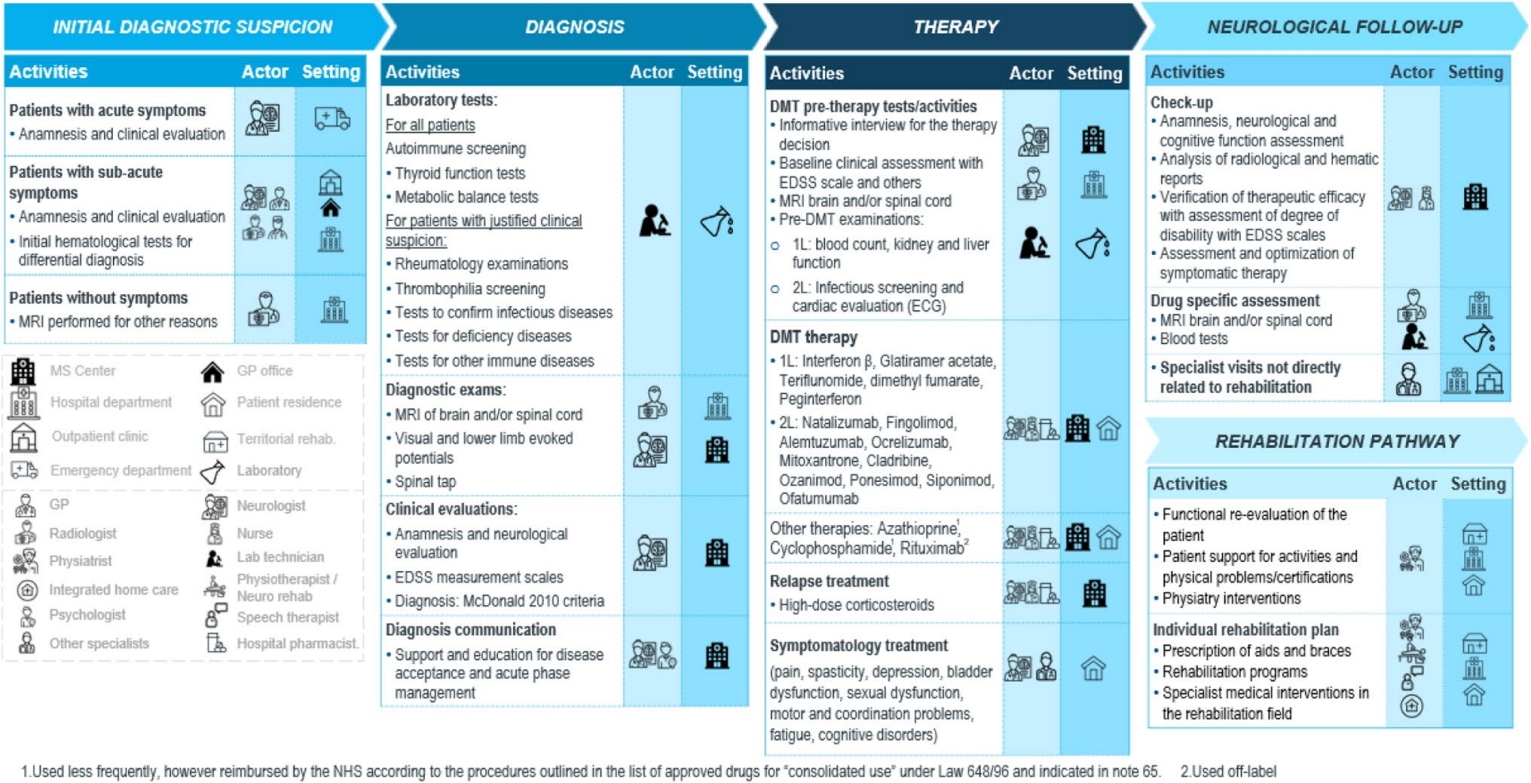
Schematic representation of the MS care delivery value chain, showing the different phases of the ICP and the relative actors and settings involved in the health services delivery

In the first phase, the current patient pathway was identified, based on the literature analysis, and the evidence was synthesized by designing the care delivery value chain (Fig. 2) and mapping the organizational flow. As illustrated in Fig. 2, the care delivery value chain identifies activities, professionals, and settings in which health services are provided across the phases of MS care. Phases include the initial diagnostic suspicion, the actual diagnosis and treatment decision, care enrollment, neurological follow-up and the rehabilitation pathway. Results show that different phases of the pathway should be delivered in different settings that are more appropriate to the health service being provided. For instance, the diagnostic phase and the neurological follow-up are carried out in the MSC or in the Neurological Department, where specialized staff with specific competences are available. On the other hand, rehabilitation services are delivered in territorial outpatient clinics, where patients undertake the rehabilitation pathway. In addition, treatment delivery varies according to the route of administration. Intravenous therapies are typically addressed in a day hospital setting, while oral and injectable therapies can be managed either at home or within outpatient clinical settings. The model promotes the provision of care in the most appropriate setting, rather than centralizing all stages within hospitals, thus striving for the integration of the MSC in the healthcare network.

In addition, expert interviews provided valuable insights into the gap between the theoretical pathway, outlined in the ICPs, and the actual operational practice of most MSCs, shedding light on common barriers and pain points within the MS pathway. For example, it was found that there is limited coordination between MSCs and local health services, as evidenced by quantitative data showing that 80% of centers lack referral protocols. This leads to delays in symptom identification and treatment definition, with only around 18% of referrals being made directly to MSCs by general practitioners (GP). Furthermore, specialists pointed out that dedicated pathways for receiving consultations from other medical specialties (e.g. gynecology, urology…) are limited, and there is limited formalization of communication channels between professionals, further complicated by the low use of digital tools. Finally, the rehabilitation pathway emerges as a critical area, given the limited availability of services in hospitals and the difficulty in identifying territorial outpatient clinics that can provide specific neurorehabilitation services, and consequently the lack of referral of pwMS from the MSC to territorial facilities. In addition, in 43% of the surveyed centers, physiatrists and physiotherapists did not have regular consultations with the neurological team to discuss the patient’s condition once they started a rehabilitation program. Interviews led to the definition of four pillars, on which the proximity model is built upon, aimed at strengthening MS management and guiding improvements in health service delivery, being: integration between the hospital and the territory, multidisciplinarity collaboration, empowerment of actors involved and digitalization.

**Fig. 3:**
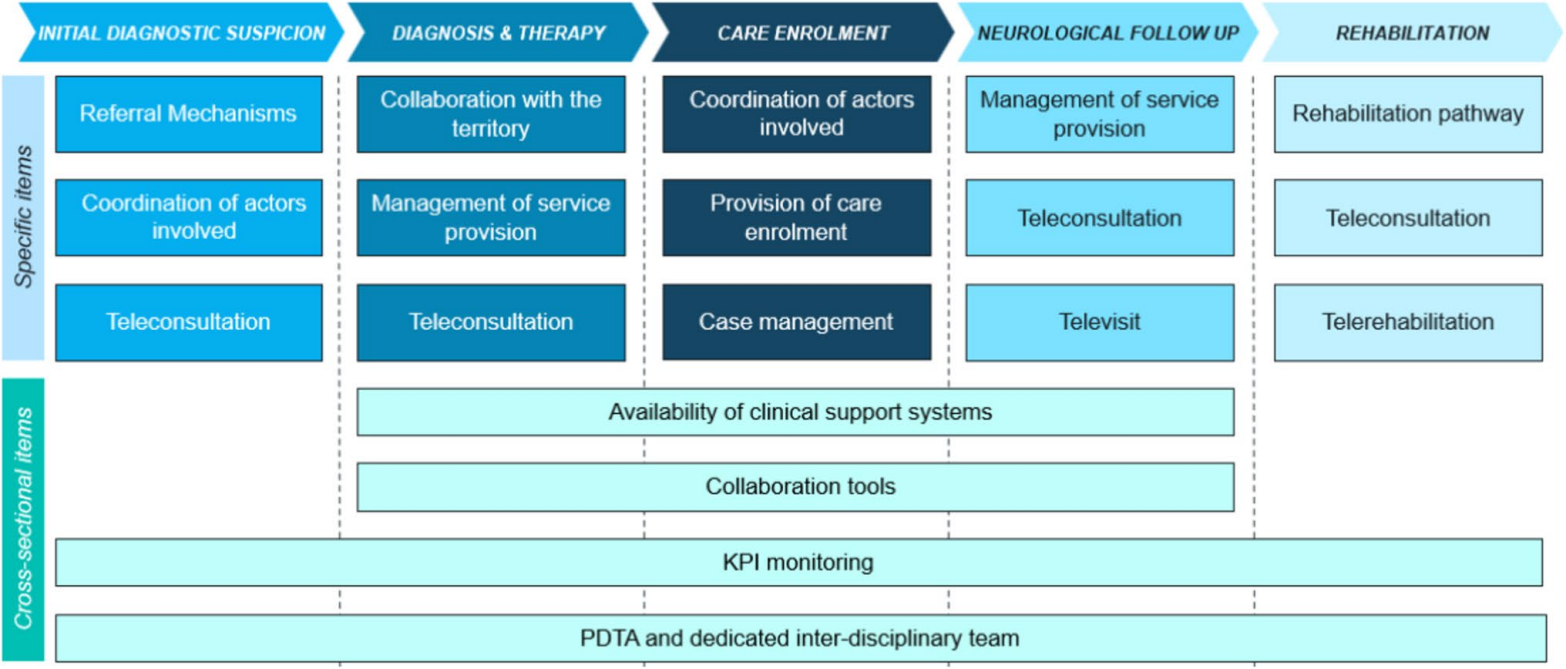
Representation of the dimensions of the Maturity Model framework. In the upper section, items related to specific phases of the ICP are reported. The lower section items are relevant for the whole pathway

The initial findings were formalized to pave the way for defining the Maturity Model framework, as previously mentioned. The goal is to evaluate various contexts and provide guidance to MSCs in enhancing the patient pathway. Figure 3 illustrates the framework, where each column represents a phase of the care journey. Within these columns, three dimensions are depicted, capturing crucial aspects essential for measuring organizational effectiveness. Moreover, the model identifies four additional overarching dimensions, which refer to the whole clinical pathway. These dimensions include the presence of a defined ICP and monitoring systems, as well as digital tools to manage and share clinical data. Each of the 19 dimensions has 4 levels of maturity, with the fourth capturing the optimal MS proximity care model (See Supplementary 3).

Finally, four process indicators were selected from the national ICP outlined by AGENAS, which are the first four KPIs reported in Supplementary 2, monitoring: the time from first contact with a neurological facility to the start of DMT therapy; the performance of at least one neurological follow-up visit per patient per year; the performance of at least one brain MRI per year for patients undergoing DMT therapy; and, finally, the number of televisits provided.

### Pilot implementation

#### Baseline results and action plan definition

The model designed in the previous phase was used to assess each of the 14 MSCs. Therefore, the theoretical pathway was localized by mapping the patient flow of each specific center, with the settings in which the activities were delivered and the professionals involved. For each phase, the center’s pathway was compared with the optimal one, highlighting positive aspects and areas of improvement. Additionally, the center was positioned on the Maturity Model, meaning that for each dimension the center was given a level from 1 to 4, allowing for a representation of the difference with the optimal pathway. This, together with the measurement of the four process KPI, gave a clear picture of the aspects that could be improved for each center. Accordingly, the four pillars defining the areas of improvement, hospital-territory integration, multidisciplinarity, actors’ empowerment and digitalization, were ranked from the most to the least urgent to assess, in order to have a guidance in the action plan definition. Table 2 illustrates, per each MSC, the three dimensions of the Maturity Model that were identified as improvement areas because of their low level, as well as the pillar that would most likely be impacted if corrective actions were taken. After the identification of improvement areas, the plan was outlined with specific actions to be implemented in a defined period and discussed with the MSC teams and tailored to their needs and criticalities. Touchpoints to monitor the implementation of the plan occurred after 3 months and 6 months from its definition, to measure improvements in KPI and Maturity Model positioning.

**Table 2:** Improvement areas identified in the 14 MSCs

MSC	Dimension 1	Dimension 2	Dimension 3	Pillar impacted
A	Collaboration with the territory	Coordination of actors involved	Management of service provision	Multidisciplinarity and hospital territory integration
B	Collaboration with the territory	Coordination of actors involved	Management of service provision	Multidisciplinarity and hospital territory integration
C	Rehabilitation pathway	Provision of care enrolment	Collaboration with the territory	Hospital territory integration
D	Rehabilitation pathway	Provision of care enrolment	Availability of clinical support systems	Hospital territory integration and digitalization
E	Availability of clinical support systems	KPI monitoring	Case management	Digitalization
F	Collaboration with the territory	Coordination of actors involved	Management of service provision	Hospital territory integration
G	Provision of care enrolment	ICP and dedicated multidisciplinary team	Coordination of actors involved	Multidisciplinarity
H	Rehabilitation pathway	Provision of care enrolment	Collaboration with the territory	Hospital territory integration
I	Collaboration with the territory	Coordination of actors involved	Management of service provision	Multidisciplinarity and hospital territory integration
J	Coordination of actors involved	Provision of care enrolment	Management of service provision	Multidisciplinarity
K	Collaboration with the territory	Availability of clinical support systems	Teleconsultation	Hospital territory integration and digitalization
L	Management of service provision	Collaboration with the territory	Provision of care enrolment	Hospital territory integration
M	Collaboration with the territory	Coordination of actors involved	Referral mechanisms	Multidisciplinarity
N	Management of service provision	Collaboration with the territory	Provision of care enrolment	Hospital territory integration

For each MSC the 3 most relevant dimensions for improvement have been reported, as well as the pillars on which the actions proposed would mostly impact

NA: information not available

#### Preliminary pre-post analysis

Preliminary results emerging from the Maturity Model analysis after 6 months from the action plan definition are reported in Table 3 (Also see Supplementary 4). Overall, results show an increase in the average positioning of centers on the Maturity Model, accordingly all the dimensions on average have had an increase in level. Both in the diagnosis and the neurological follow-up, there is a relevant increase in management of service provisions, counting an average percentage increase of, respectively, 110% and 115%. This dimension primarily captures the accessibility to diagnostic tests, mainly MRIs, therefore considering the availability of dedicated slots within the MSC, or the hospital, and collaborations with the territory for the external execution of MRIs. The dimension that had the lowest average increase is telerehabilitation, maintaining its low position in both periods, meaning that in most contexts there is no implementation of telemedicine in rehabilitation services. Another aspect worth underlining is the average level of 3 in the second period for the case management, referring to the fact that most centers have a proactive management of the care pathway, optimizing hospital access and supporting patients in programming visits and exams. These activities are performed prevalently by MS nurses or a dedicated MS case manager.

**Table 3:** Maturity model preliminary pre-post analysis

Phase	Dimension	MSCs average score at T1	MSCs average score at T2	MSCs average Δ %
Initial diagnostic suspicion	Referral mechanisms	1.0	1.83	+ 83
	Coordination of actors involved	1.42	1.83	+ 29
	Teleconsultation	1.33	1.92	+ 44
Diagnosis	Collaboration with the territory	1.42	1.92	+ 35
	Management of service provision	1.67	3.5	+ 110
	Teleconsultation	1.25	2.17	+ 73
Care enrolment	Coordination of actors involved	1.5	2.17	+ 44
	Provision of care enrolment	2.0	2.5	+ 25
	Case management	2.25	3.0	+ 33
Neurological follow-up	Management of service provision	1.67	3.58	+ 115
	Teleconsultation	1.25	2.0	+ 60
	Televisit	1.58	1.75	+ 11
Rehabilitation pathway	Rehabilitation pathway	2.0	2.25	+ 13
	Teleconsultation	1.17	1.67	+ 43
	Telerehabilitation	1.0	1.08	+ 8
Cross-sectional items	Availability of clinical support systems	1.5	1.83	+ 22
	Collaboration tools	1.17	1.67	+ 43
	KPI monitoring	1.0	1.75	+ 75
	ICP and dedicated multidisciplinary team	1.83	2.67	+ 45

Data consider the average score of 11 MSCs, because 3 MSCs were not included in the analysis as the minimum period of 6 months from the action plan definition had not yet passed. Centers rated each dimension from 1 to 4 through a self-assessment form, with 1 being lowest and 4 highest. T1 represents the value of the average scores of MSCs for each dimension at the baseline and T2 represents the value after at least 6 months from the action plan definition. The last column reports the percentage change of the average level of each dimension in the 2 periods. The average Δ % is measured by calculating the average percentage change of the mean value for each dimension: (MSCs average score in T2 – MSCs average score in T1)/MSCs average score in T1

### Model validation

An initial set of 25 KPIs and 19 qualitative dimensions were proposed to the panel. An additional KPI and dimension were suggested, leading to 26 KPIs (Supplementary 5) and 20 dimensions (Supplementary 6) to vote for in the first round. After the first round, consensus was reached for 4 KPIs, meaning that 75% of panelists rated them 4 or 5 on the Likert scale for 3 of the 4 criteria. Three of them were KPIs measured in the pilot phase and reported in the AGENAS ICP: the amount of time between the first contact with a neurology facility and the start of DMT therapy, the execution of at least one neurological follow-up visit per patient per year, the execution of at least one brain MRI per year for patient in therapy with DMT. The remaining 22 KPIs were newly voted, exhibiting the inclusion of 5 additional KPIs, for a total of 9 validated KPIs, reported in Table 4 (Also see Supplementary 5), referring to different aspects of the patient pathways such as: diagnostic activities, progression of the condition, rehabilitation pathway, follow-up visits, care complexity, appropriate settings and multidisciplinarity.

**Table 4:** KPIs validated by the Delphi method

Area	Type	Indicator	Numerator	Denominator
Appropriate accesses/setting	Process	% Inappropriate accesses to the ER (patients enrolled in the MS Center with accesses to the ER)	Number of inappropriate accesses to the ER of MS patients enrolled in the MSC	Number of accesses to the ER of enrolled patients
Care complexity	Volume	% Patients enrolled with high care complexity	% Patients with EDSS > 6.5	Total patients enrolled
Care complexity	Volume	% MS patients in therapy at the MS center (calculated for I and II line)	Patients on 1L therapy Patients on 2L therapy	Total patients enrolled
Adequate follow-up frequency	Process	Undergoing at least one neurological visit/patient/year	Number of patients diagnosed with MS who have undergone at least one visit in the year under review	Total patients enrolled
Multidisciplinary approach	Process	Level of multidisciplinary care enrolment	Total scheduled services with other specialists	Total services performed with other specialists
Rehabilitation pathway	Process	% MS patients treated in inpatient rehabilitation services (in different settings) and territorial rehabilitation services	Number of patients treated by rehabilitation services	Total patients enrolled
Diagnostic examination	Process	Performing at least one brain MRI per year for all patients on DMT therapy	Number of patients diagnosed with MS on DMT therapy who had at least one brain MRI in the year under review	Number of MS patients on DMT therapy
Diagnostic examination	Process	Time between first contact with a neurology facility and initiation of DMT therapy	Number of patients newly diagnosed with MS in the year under review who started DMT within 90 days of first contact with the neurological structure in the same year	Part of the incident cohort that started a DMT from April 1 to December 31
Disease progression, complications, adverse events	Outcome	% Patients hospitalized for infectious complications	Number of hospitalizations, non-rehabilitation, in the year under review with a primary or secondary discharge diagnosis of MS with one or more of the following diagnoses (pneumonia, urinary tract infection)	Total patients enrolled

A total of 9 KPI were validated in the 2 rounds. The table represents the area of the patient pathway considered, the type of KPI, the name of the indicator and how it is calculated

Referring to the Maturity Model, in the first round 9 dimensions out of 20 were included, therefore reaching rates of 4 or 5 from more than 75% of panelists. The remaining dimensions were revoted in the second round leading to the inclusion of 17 dimensions and the exclusion of 3, as reported in Supplementary 6. Excluded dimensions are related to referral mechanisms in the initial diagnostic suspects, the use of telemedicine in the rehabilitation phase and tool for collaboration, for instance digital integrated systems for sharing clinical data or telemedicine platforms for teleconsultations.

## Discussion

Addressing chronic disease management is a pressing challenge for healthcare systems worldwide, given the aging population and the rise in long-term conditions that require continuous care [28]. Most counties have developed their systems around acute hospital care, rather than providing organizational models to support chronicity, therefore calling for a new paradigm shift towards a system that considers chronic patients and their needs [29, 30]. After the pandemic, Italy has seen an ongoing transformation in this direction, with significant policy changes, aiming to support novel care models through the establishment of new intermediate care settings and enhance primary and out-of-hospital care [7, 8]. Today, MS care prevalently revolves around hospital settings and more specifically in Italy in MSCs. However, there is an urge in most European countries to reduce hospital access and integrate the care provision across various settings to enhance efficiency and effectiveness [31, 32]. Despite the importance of integrated care among different care settings and specialists, the MSC maintains its central role within MS management due to the complexity of the disease and the need of specialized staff to assess, monitor and support the pwMS along his or her care journey [33–35]. DMTs play a fundamental role in slowing down MS progression, and these therapies require the supervision of a specialized team, confirming the essential role of MSCs [34]. However, in order to reduce organizational complexities and optimize hospital capacity, there’s a growing acknowledgment within the healthcare landscape for greater territorial integration [31]. The current model is characterized by fragmentation and segmentation, resulting in the inefficient use of healthcare resources, necessitating improved coordination among various professionals and healthcare settings [36–38]. In this context, the project stands as a practical example of a proximity care that advocates for a holistic approach to MS management beyond the traditional care delivery model.

The proposed proximity care model builds upon four pillars that guide improvements in MS management, being: integration between the hospital and the territory, multidisciplinarity collaboration, empowerment of actors involved and digitalization. To guide the implementation of these pillars, a Maturity Model framework was designed to monitor their integration in the MS pathway. Furthermore, nine KPIs have been validated to be part of the model, to evaluate the impact of adopting the new model in terms of organizational indicators.

In existing literature, there is no unique model of integration defining coordination among different healthcare facilities and organizations. European countries have experimented with different approaches, tailored to their healthcare systems, aimed at restructuring chronic care delivery to achieve greater feasibility and sustainability. Two main approaches emerge: a top-down approach, which is orchestrated at the national level, leveraging regulations and funding, and a bottom-up approach, which relies on regional and local initiatives [31]. Despite the recognition of integrated care models as effective solutions for overcoming chronic care challenges, the application and evaluation of these models in MS remains relatively limited [39]. However, it is relevant to address this gap, given the significant impact of the disease. For instance, the World Health Organization (WHO) has emphasized the importance of establishing comprehensive care pathways for neurological conditions. These pathways should integrate multiple levels of care, promoting continuity of care between providers and health system tiers through effective referral and communication mechanisms [40]. Therefore, confirming the relevance of this pillar in the newly defined model.

The second pillar emphasizes the crucial role of multidisciplinarity in addressing the complexities of MS care [41]. It has been acknowledged in literature that effective management of symptoms of complex chronic diseases, such as MS, relies on the collaboration among various healthcare specialists, highlighting the pivotal role of multidisciplinary teams in ensuring a holistic patient support throughout the care journey [42–44]. However, organizing multidisciplinary care poses challenges, due to limited specialists and high caseloads [43], necessitating the establishment of formalized protocols to optimize collaboration. Sorensen et al. have outlined the ideal composition of the MS multidisciplinary team, typically consisting of a neurologist specialized in MS and a specialist nurse as the core components. Additionally, the inclusion of several specialists among the care pathway such as neuropsychologists, specialist in neurological rehabilitation (speech, occupational therapist), neuro-radiologists, psychologists and social workers contribute to comprehensive care delivery [34]. Moreover, considering the variety of symptoms associated to MS, it would be beneficial to consider collaborations with neighboring specialties such as neuro-ophthalmologists and urologist throughout the pathway [34, 44].

It is important to highlight that the development of the Maturity Model included the dimension of Palliative Care. In light of recent EAN guidelines [45], the StayHome project has surpassed current care models, proposing improvements in the management of highly disabled pwMS that promote their values and preferences in end-of-life decisions, which involve complex ethical implications. The role of MSCs in offering advance care planning and tools to ensure patient autonomy, such as the Shared Care Planning (in the Italian text “Pianificazione condivisa delle cure”) provided for by the Italian legislation (Law n. 219/2017 “Norme in materia di consenso informato e di disposizioni anticipate di trattamento” [Provisions for informed consent and advance directives] [46]. Palliative Care should be understood as an "early and simultaneous" approach, as stated by the WHO, and can be generalist or specialist [47]. Generalist care involves developing palliative skills among neurologists and neurorehabilitators, while specialist care requires full integration of neurology, rehabilitation, and specialized palliative services to provide a comprehensive, efficient, and person-centered care system throughout the entire trajectory of MS. By focusing on neuropalliative care, the StayHome project has completed the aforementioned model of Sorensen et al. [34].

As an integral part of the multidisciplinary team, the professional case manager plays a vital role for both the patient’s experience and the healthcare organization [48], determining the third pillar of the model. The case manager guides the team’s effort by overseeing, measuring, and assessing outcomes achieved, aiming for specific goals, such as reducing utilization of scarce resources and controlling costs [48, 49]. A Canadian study found that chronic patients in primary care considered care manager nurses as their main point of reference and contact, relying on them for guidance and support. Case managers actively engaged patients in developing and implementing individual service plans, streamlining access to healthcare services and information, and facilitating seamless transitions between professionals and care settings [50]. The value of integrating case management into care pathways has also been validated in the context of MS [51], mainly in the UK where specialist MS nurses have become pivotal figures in MS management [51–53]. They have a wide range of functions, with significant involvement in clinical decisions and have the capacity to oversee the entirety of patient care services, distinguishing their role from that in other European countries [54]. However, the case manager’s main role in most health contexts is orchestrating the care provision and enhancing collaboration among actors, aiming to minimize unnecessary care access and ensure continuity of care [54].

The fourth and last pillar of the model is digitalization. The unique circumstances of the Covid-19 pandemic provided the opportunity to introduce telemedicine resources as a solution to scarce resources and to limit patient access to hospitals, thereby reducing the exposure to infection risks [55]. However, the use of telemedicine extends beyond the pandemic, serving as a valuable means of reaching patients, contingent upon the presence of digital infrastructure [55]. Telemedicine offers time and cost savings for both patients and healthcare providers, while also enhancing efficiency in hospital and clinic workflows due to its rapid and advantageous nature [56–58]. Several studies have found that patients generally respond positively to the use of digital tools, showing high levels of satisfaction and acceptance. This is largely attributed to the convenience of avoiding travel expenses, reducing missed workdays, and saving time [55, 57, 59]. Additionally, telemedicine can play a role in facilitating digitally shared clinical data and teleconsultations among healthcare professionals to reduce time dedicated to multidisciplinary collaboration [39], as recently acknowledged by the WHO [40]. Other aspects of telemedicine, such as telemonitoring and telerehabilitation, represent promising avenues for future development in optimizing care delivery [60]. Specifically for MS, telemedicine has emerged as a viable solution for bridging gaps in the MS pathway caused by distance and disability [61]. While it has shown technical feasibility, widespread adoption faces challenges such as the need for adequate infrastructure for both healthcare providers and patients, as well as limited or no reimbursement for remote services, which varies among and within countries [55, 61]. Addressing these issues requires further attention by healthcare policymakers.

As discussed, the Maturity Model and KPIs serve as crucial tools for guiding centers in the application of the proximity care model. However, to ensure their effectiveness, the monitoring system should become systematic, enabling continuous evaluation of the actions taken to enhance model adoption. One approach MSCs could implement is the integration of organizational components of the Maturity Model and relevant KPIs into formalized integrated patient care pathways, involving specific territorial areas including both the MSC and other territorial facilities [62]. These formalized pathways require regular auditing and feedback, therefore progress could be monitored to ensure effective implementation of the model. Another potential strategy lies within embedding the model’s characteristics in the health organization’s integrated plan for activities and organization (referred to in Italy as PIAO), where strategic and operational objectives are defined, along with indicators and metrics for performance evaluation. Through the performance report, health organizations are required to provide results achieved every two years based on the plan and would include the evaluation of the model adoption. To achieve a more comprehensive implementation that encompasses both hospital and territorial facilities, the MS proximity care model could be integrated into regional health plans rather than limiting it to individual health organizations. Lastly, in order to establish a national benchmarking on MSC’s, key aspects of the proximity model could be investigated through an annual survey. Since the AISM Barometer already offers valuable insights into various aspects of MS care, the survey results could be included as well, providing an overview of the extent of integrated care offered to MS patients across Italy.

This research sees some methodological limitations, such as the partial geographical representation of Italian regions in the pilot study. To provide a more comprehensive national perspective, involving one MSC per region could have been beneficial, considering the high heterogeneity in healthcare organizational models. However, the selected centers are evenly distributed on the national territory, with representatives of northern, central, and southern Italy.

Another important limitation concerns the calculation of KPIs. Even though the AGENAS ICP defines calculation protocols for the four selected KPIs, centers have encountered difficulties in accessing and retrieving data from the indicated data streams, with limited possibility of automatic calculations. Furthermore, some data streams encompass territorial facilities without MSCs having direct access to related information, complicating the calculation process. For example, while many MRI tests are provided by territorial diagnostic centers, the MSCs often do not have access to the precise number of patients that underwent the examinations. Therefore, KPIs were not included in the preliminary pre-post analysis. However, Italy’s ongoing digital transformation holds promise for improved data accessibility, with MSCs currently undergoing information systems development to facilitate automated KPI calculation in the future.

While patients were not directly included in the model definition, AISM was actively involved throughout the project to ensure the inclusion of a patient perspective on MS management. AISM is engaged in addressing all aspects related to MS in a structured and organic manner, operating through 98 provincial sections, 38 local operative groups, and over 14,000 volunteers. Furthermore, the association publishes an annual MS Barometer, combining patient stories with real-world data retrieved from the MS Registry, providing a view on the disease and insights into living with the condition. This contribution is widely recognized by policymakers as key to gain a comprehensive understanding of the MS landscape [63]. Therefore, the defined proximity model primarily reflects physicians’ experiences as well as an aggregated patient perspective through AISM’s participation. However, given that MS is a chronic, lifelong condition, incorporating the direct patient input could be a valuable evolution of the model. This could be achieved by refining the Maturity Model to include patient-centered dimensions and by including patient-reported outcomes and experience measures, providing a more comprehensive approach, ultimately leading to a more patient-centered MS management model.

## Conclusions

In conclusion, for the first time in Italy, a proximity care model has been defined for MS care. The model definition followed a bottom-up approach, taking into account regional and local specificities, with the collaboration of healthcare professionals and patient associations, determining its scalability at the national level. The project has had an impact on over 150 healthcare professionals and 16,000 patients, supporting an initial paradigm shift to a higher territorial integration, which could be adopted by other MSCs.

## Supplementary Information

Below is the link to the electronic supplementary material.

Supplementary file1 (DOCX 168 KB)

## Data Availability

The dataset used and analyzed during the current study is available from the corresponding author upon request to qualified researchers (i.e., affiliated to a university or research institution/hospital).
