# Supplementary material for: Implementing proximity care for people with multiple sclerosis in Italy: the bottom-up approach of the StayHome project
Source: J Neurol. 2025 Jan 7;272(1):96. doi: 10.1007/s00415-024-12749-8 (PMC11706871; doi:10.1007/s00415-024-12749-8)
Supplement: Supplementary file 1 — Supplementary file1 (DOCX 168 KB) [file 415_2024_12749_MOESM1_ESM.docx]

**Supplementary Information**

**Supplementary 1**

CAWI Questionnaire addressed to 40 physicians investigating the different phases of MS management in their practice.

Thank you Doctor. Before we begin the interview, some brief introductory questions.

**E1. Does the facility where you work deal with the treatment and/or rehabilitation of patients with Multiple Sclerosis?**

1. Yes
2. No *-> thank you and close*

**E2. Are you personally involved in the treatment and/or rehabilitation of patients with Multiple Sclerosis?**

1. Yes
2. No *🡪 if 'NO' thank and ask for another colleague who cares for patients with Multiple Sclerosis*

**E3. Done 100 your professional activities, how much of your time is devoted to patients with Multiple Sclerosis?**

%

*🡪 if <20%, thank and close*

**E4. Do you practice as...?**

1. Neurologist
2. Physiatrist
3. Neurological rehabilitator
4. Other specialty

*🡪 if other specialty, thank and close*

*Code 1: Neurologist.*

*Code 2-3: Physiatrist-Rehabilitation.*

**E5. What is your specialty?**

1. Neurology
2. Physiatrics
3. Neurorehabilitation
4. Other (specify: )

**E6. What is the name of the department in which you work?**

***0. GENERAL FRAMEWORK***

**D1. Doctor, where do you carry out your predominant activity in the treatment of patients with Multiple Sclerosis?**

(*single answer*)

1. MS Center
2. Neurology Department of Non-SM Centers
3. Department of Physiatrics of Non-SM Centers
4. Neuro-rehabilitation/rehabilitation department of non-SM Centers
5. Day Hospital
6. Other hospital departments (please specify )
7. Territorial outpatient clinic (including rehabilitation centers)
8. Patient's domicile

**D2a. In the center at which you work, do you conduct rehabilitation follow-up activities of patients with Multiple Sclerosis? (single answer)**

1. Yes
2. No

If yes to d2a

**D2b. Where are the rehabilitation follow-up activities of patients with Multiple Sclerosis carried out? (e.g., visits and rehabilitation activities) (multiple answer)**

1. Activities take place within the SM Center.
2. Activities take place at the neurology department
3. Activities take place at the department of physiatrics
4. Activities take place at the neurorehabilitation department
5. Other ( )

If not to d2a

### **D2c. To the best of your knowledge, are indications provided to MS patients about facilities and arrangements regarding rehabilitation follow-up activities? (single answer)**

1. Yes, a list of contracted facilities is provided where the patient can perform follow-up rehabilitation activities
2. No, the management of this aspect is left to the free initiative of the patient
3. Can't answer

### **D3. What % of MS patients are usually accompanied by a caregiver? In answering please refer to pre-pandemic normal practice, i.e., do not consider situations particularly impacted by the COVID-19 emergency.**

% patients accompanied by caregivers

*If answer [>0] in D3 -> ask question D3a:*

### **D3a. What % of these caregivers turn out to be of working age?**

% caregivers of working age

***1.CLINICAL MANAGEMENT***

***1.1 CENTER FRAMING AND PATHOLOGY MANAGEMENT***

Doctor, let's talk about the case history of **patients with Multiple Sclerosis in charge of the facility where you work.**

For our purposes, it is important that you consider the total number of patients followed by the department/ambulatory at which you work, not just those you personally see.

By ward we mean both inpatient, outpatient, and day hospital.

### **D4.** **How many Multiple Sclerosis patients does the center/department in which you operate have charge of? In stating this number please ...**

- - Consider **usual/normal activity**, i.e., do not consider periods particularly impacted by the pandemic

### Take into account all patients in your department's care, including day hospital and outpatient (no personal caseload) for multiple sclerosis

- - Think of all patients seen, both those on medication and those who have not yet started treatment as well as those on momentary withdrawal of therapy. Also consider patients you do not see often but who are still in your department's care.

No. patients in charge of the center/department in which it operates

**D5. Of all ... [d4] patients with Multiple Sclerosis in your department's care, how many have a form....**

|

| a) CIS (clinically isolated syndrome) | \| | \| \| |
| --- | --- | --- |
| b) RR (relapsing remitting) | \| | \| \| |
| c) SP (secondarily progressive) | \| | \| \| |
| d) PP (primarily progressive) | \|\| | \| |

|

|

Tot = d4

**D6. How many new patients with Multiple Sclerosis are diagnosed in a year at your Center?**

No. new patients diagnosed in one year in the center where it operates

*If target = NEUROLOGIST -> ask for Section* ***1.2 diagnosis and diagnostic suspect****; otherwise skip to Section* ***1.3 Treatment*** *( )*

***1.2 DIAGNOSIS AND DIAGNOSTIC SUSPECT***

### **D7. Made 100 patients in charge of your department, how many of them were referred to your department with SUSPECT OF MULTIPLE SCLEROSIS performed by. (multiple answer)**

| 1. GP | \| \| \| \|% |
| --- | --- |
| 2. Territorial neurologist | \| \| \| \|% |
| 3. Hospital neurologist | \| \| \| \|% |
| 4. PS physician | \| \| \| \|% |
| 5. Ophthalmologist | \| \| \| \|% |
| 6. Orthopedic | \| \| \| \|% |
| 7. Other (specify: ) | \| \| \| \|% |
|  | tot=100% |

If at d7 cited code 1 or 3 or 5 >0 (GP, territorial neurologist or ophthalmologist)

### **D8. In what ways is the patient with suspected Multiple Sclerosis primarily referred by the territorial doctor to the center at which he or she works? (multiple answer)**

1. Reservation through CUP
2. Direct contact with the MS Center or its department (e.g., email or telephone contact)
3. Other (specify: )
4. I don't know.

If at d7 cited code 1 or 3 or 5 >0 (GP, territorial neurologist or ophthalmologist)

### **D9. Think about the arrival of MS patients to your department from territorial medicine (both GPs and other territorial specialists). Is there a codified protocol on the arrival of MS patients to your center, or is the referral to your center based on knowledge between clinicians? (single answer)**

- - There is a codified referral protocol
  - Knowledge among clinicians
  - Other (specify: )

***1.3 TREATMENT, ADDITIONAL SERVICES AND PATIENT SUPPORT PROGRAM***

Doctor, in completing the following section of the questionnaire, consider a "normal" post-pandemic Covid- 19 scenario.

*To all*

**D10**. **Which of the following additional services are present in your Center?** (*multiple answer*)

1. Direct telephone contact with dedicated staff
2. Psychological support
3. 24-hour emergency access
4. Continuous presence patient association
5. Platform/app for reservations and visit reminders
6. Support desk for administrative practices (applications for exemptions and other certificates)
7. Other ( )
8. None
9. Can't answer

### **D11. What Patient Support Program services (sponsored by pharmaceutical partner associated with specific therapeutic proposal) does your Center use to date? (multiple answer)**

1. Home delivery of the drug
2. Home educational support to pathology, product and self-administration
3. Remote educational support to pathology, product and self-administration
4. Support for proper use and for any issues related to devices and consumables
5. Remote reminder for appointments at the Center
6. Home monitoring of hematological parameters
7. Rehabilitation treatments at home
8. Home infusion DMT treatments
9. None
10. Other ( )
11. Can't answer

### **D12. In your opinion, which Patient Support Program services offer the most value to the patient?**

*(multiple answer)*

**D12a. Which one most?** *(single answer)*

1. Home delivery of the drug
2. Home educational support to pathology, product and self-administration
3. Remote educational support to pathology, product and self-administration
4. Support for proper use and for any issues with devices and consumables
5. Remote reminder for appointments at the Center
6. Home monitoring of hematochemical parameters
7. Rehabilitation treatments at home
8. Infusion treatments at home
9. None
10. Other ( )
11. Can't answer

### **D13. Does your center have a case manager for the management of the MS patient?** *(single answer)*

1. No
2. Yes, role filled by neurologist
3. Yes, role filled by nursing staff
4. Yes, role filled by physiatrist
5. Yes, role held by other figure (specify )
6. Can't answer

### **D14. Are you personally involved in the clinical follow-up of patients with Multiple Sclerosis?** *(single answer)*

1. Yes
2. No

*If answer [1] in D14 -> ask section* ***1.5 clinical follow-up****; otherwise skip to* ***question D19***

***1.5 CLINICAL FOLLOW-UP***

### **D15. What are the main channels through which a patient can book a clinical follow-up visit?** *(multiple answer)*

1. Preferential channel dedicated to Multiple Sclerosis patients (e.g., dedicated email or app, phone contact with dedicated doctor/nurse)
2. Informal direct contact with physician/nurse practitioner in the center or department
3. Regional CUP
4. Can't answer

*If code 1 to d15*

### **D15a. Which preferential channel dedicated to patients with Multiple Sclerosis exist?** *(multiple answer)*

- 1. Dedicated mail
  2. Dedicated app
  3. Telephone contact with dedicated health personnel
  4. Other (specify: )

### **D16. Does your center have a multidisciplinary team of specialists who deal specifically with the clinical management of the patient with Multiple Sclerosis and who schedule regular, structured meetings for the discussion of clinical cases?** *(single answer)*

1. Yes
2. No

*If answer [1] in D16 -> ask question D16a*

**D16a. Which figures are part of this multidisciplinary team?** (*multiple answer*)

1. Neurologist
2. Infectivologist
3. Cardiologist
4. Psychologist
5. Psychiatrist
6. Physiotherapist
7. Physiatrist
8. Radiologist
9. Other specialist (specify )
10. Can't answer

*If answer [2] in D16 -> ask question D16b*

### **Q16b. Which figures are involved in the clinical follow-up of the patient with MS?** (multiple answer)

1. Neurologist
2. Infectivologist
3. Cardiologist
4. Psychologist
5. Psychiatrist
6. Physiotherapist
7. Physiatrist
8. Radiologist
9. Other specialist (specify )
10. Can't answer

### **D17. What additional professionals are called upon by the neurologist for consultations when needed?**

*(Multiple answer)*

1. Endocrinologist
2. Infectivologist
3. Cardiologist
4. Gastroenterologist
5. Psychologist
6. Psychiatrist
7. Physiotherapist
8. Physiatrist
9. Radiologist
10. Other specialist (specify )
11. Can't answer

### **D18. What clinical follow-up activities are performed to date at the patient's home using remote modalities as well?**

(*Multiple answer*)

1. None
2. Blood samples
3. Televisitis
4. Support for obtaining certificates
5. Counseling activities by other specialists
6. Disability assessment/patient function (specify scales used )
7. Cognitive assessment and remote neurological testing (specify scales used )
8. Other (please specify )
9. Can't answer

### **D19. In your opinion, what clinical follow-up activities could be carried out in the home setting in the future using remote modalities as well?** *(Multiple answer)*

1. None
2. Blood samples
3. Televisitis
4. Support for obtaining certificates
5. Counseling activities by other specialists
6. Patient disability/functionality assessment (specify scales to be used )
7. Cognitive assessment and remote neurological testing (specify scales to be used )
8. Other (please specify )
9. Can't answer

***1.6 REHABILITATION FOLLOW-UP***

*To all*

### **D20. Doctor, let us now discuss the rehabilitation follow-up of patients with Multiple Sclerosis. Which professional figure determines the level of rehabilitation follow-up that will follow the patient?** *(Single answer)*

1. Neurologist
2. Physiatrist
3. Neurorehabilitator
4. Other (please specify )

### **D21. Are there touchpoints or alignment meetings between the Neurologist in charge of setting and monitoring drug therapy (clinical follow-up) of MS patients and the specialist in charge of rehabilitation follow-up?** *(single answer)*

1. Yes, through regular and codified meetings (both in person and remotely)
2. Yes, through informal and uncoded meetings (both in person and remotely)
3. No alignment meetings are scheduled

*If d21 code 1 or 2*

### **D21a. And on average, how many times in 1 month are these alignment meetings held between the Neurologist in charge of setting and monitoring drug therapy (clinical follow-up) of MS patients and the specialist in charge of rehabilitation follow-up?**

**| | |** nr of times alignment meetings are held in a month

### **D22. Doctor, which of these answers most closely resembles the activities you perform in the rehabilitation follow-up of patients with Multiple Sclerosis?** *(single answer)*

1. I am involved EXCLUSIVELY in clinical follow-up and I am NOT involved in the rehabilitation follow-up of the patient
2. I mainly do clinical follow-up and provide general guidance on the rehabilitation follow-up activities to be performed by the patient
3. I provide specific guidance on rehabilitation follow-up activities, prescribing and detailing necessary activities, possibly activating the PRI (Individual Rehabilitation Plan)
4. Predominantly receive and partially adapt to the patient's needs rehabilitation indications provided by other specialists

- *If answer [1] in D22,* ***terminate***
- *If answer [2] in D22, ask only section* ***2.1 Rehabilitation management***
- *If answer [3] or [4] in D22, ask for both section* ***2.1 rehabilitation management and section 2.2 rehabilitation management-detail***

***2.1 REHABILITATION MANAGEMENT***

### **D23. Is there a rehabilitation team that manages the patient's rehabilitation follow-up?** *(single answer)*

1. Yes
2. No
3. Can't answer

*If answer [1] in D23 -> ask question D23a*

### **D23a. Which figures are part of this multidisciplinary rehabilitation team in the center at which you work?**

(*multiple answer*)

1. Physiatrist
2. Phoniatrist
3. Physiotherapist
4. Speech therapist
5. Occupational therapist
6. Psychologist
7. Rehabilitation nurse
8. Other (please specify )
9. Can't answer

*If answer [2] in D23 -> ask questions D23b and D23c*

### **D23b. Which professionals are involved in the rehabilitation follow-up of the patient with Multiple Sclerosis in the center at which you work?** *(Multiple answer)*

- 1. Physiatrist
  2. Phoniatrist
  3. Physiotherapist
  4. Speech therapist
  5. Occupational therapist
  6. Psychologist
  7. Rehabilitation nurse
  8. Other (please specify )
  9. Can't answer

### **D23c. To the best of your knowledge, which professionals are involved in the rehabilitation follow-up of the patient with Multiple Sclerosis at the territorial level?** *(Multiple answer)*

**D23d. And again to the best of your knowledge, what figures are involved in rehabilitation follow-up at the home level?** (*Multiple answer*)

|  | D23c | D23d |
| --- | --- | --- |
| 1. Physiatrist | □ | □ |
| 2. Phoniatrist | □ | □ |
| 3. Physiotherapist | □ | □ |
| 4. Speech therapist | □ | □ |
| 5. Occupational therapist | □ | □ |
| 6. Psychologist | □ | □ |
| 7. Rehabilitation nurse | □ | □ |
| 1. Other (specify: ) 2. Don't know | □ | □ |
|  | □ | □ |

***2.2 REHABILITATION MANAGEMENT - DEEPENING***

In this section we will elaborate on the peculiarities of rehabilitation follow-up for two macro-groups of patients according to their degree of disability on the EDSS scale.

*Referring to patients with EDSS <6.5 ... (keep text in top view for questions 24 to 26)*

### **D24. What activities are generally included in the rehabilitation follow-up for this type of patient?** *(multiple answer)*

1. Functional reassessment of the patient
2. Patient support for carrying out physical activity and sports
3. Physiatric interventions as infiltrative and symptomatic therapies
4. Programs for balance, sensory and cognitive disorders
5. Instrumental assessment of gait, balance, and posture
6. Rehabilitation activities without the use of rehabilitation machines
7. Rehabilitation activities using rehabilitation machines
8. Specialist consultation required (specify specialist )
9. Patient/caregiver educational support for prevention of worsening spasticity
10. Other (please specify )
11. Can't answer
12. None of these

### **D25. Which of these activities are currently carried out in the home setting for this type of patient?** (*multiple answer*)

1. Functional reassessment of the patient
2. Patient support for carrying out physical activity and sports
3. Physiatric interventions as infiltrative and symptomatic therapies
4. Programs for balance, sensory and cognitive disorders
5. Instrumental assessment of gait, balance, and posture
6. Rehabilitation activities without the use of rehabilitation machines
7. Rehabilitation activities using rehabilitation machines
8. Request specialist consultation (specify specialist )
9. Other (please specify )
10. Can't answer
11. None of these

### **D26. Which of these activities could be carried out in the home setting in the future, so they have the highest degree of ease of implementation?** *(multiple answer)*

1. Functional reassessment of the patient
2. Patient support for carrying out physical activity and sports
3. Physiatric interventions as infiltrative and symptomatic therapies
4. Programs for balance, sensory and cognitive disorders
5. Instrumental assessment of gait, balance, and posture
6. Rehabilitation activities without the use of rehabilitation machines
7. Rehabilitation activities using rehabilitation machines
8. Specialist consultation required (specify specialist )
9. Other (please specify )
10. Can't answer
11. None of these

### **D26.1 And which of these activities have the highest added value for this type of patient (patients with EDSS <6.5)?** (*multiple answer*)

1. Functional reassessment of the patient
2. Patient support for carrying out physical activity and sports
3. Physiatric interventions as infiltrative and symptomatic therapies
4. Programs for balance, sensory and cognitive disorders
5. Instrumental assessment of gait, balance, and posture
6. Rehabilitation activities without the use of rehabilitation machines
7. Rehabilitation activities using rehabilitation machines
8. Specialist consultation required (specify specialist )
9. Other (please specify )
10. Can't answer
11. None of these

*Referring instead to patients with EDSS >6.5 ... (keep the text in view above for questions D27 to 33)*

### **D27. What activities are generally included in the rehabilitation follow-up for this type of patient?** *(multiple answer)*

1. Functional reassessment of the patient
2. Physiatric interventions as infiltrative and symptomatic therapies
3. Programs for balance, sensory and cognitive disorders
4. Instrumental assessment of gait, balance, and posture
5. Rehabilitation activities without the use of rehabilitation machines
6. Rehabilitation activities using rehabilitation machines
7. Specialist consultation required (specify specialist )
8. Patient/caregiver educational support for prevention of worsening spasticity
9. Treatment and prevention of secondary complications
10. Assessment and management of neuropathic and nociceptive pain
11. Instrumental and clinical evaluation for use of tools such as baclofen pump or functional bandages
12. Respiratory re-education activities
13. Other (please specify )
14. Can't answer
15. None of these

### **D28. Which of these activities are currently carried out in the home setting for this type of patient?** (*multiple answer*)

1. Functional reassessment of the patient
2. Physiatric interventions as infiltrative and symptomatic therapies
3. Programs for balance, sensory and cognitive disorders
4. Instrumental assessment of gait, balance, and posture
5. Rehabilitation activities without the use of rehabilitation machines
6. Rehabilitation activities using rehabilitation machines
7. Request specialist consultation (specify specialist )
8. Treatment and prevention of secondary complications
9. Assessment and management of neuropathic and nociceptive pain
10. Instrumental and clinical evaluation for use of tools such as baclofen pump or functional bandages
11. Respiratory re-education activities
12. Other (please specify )
13. Can't answer
14. None of these

### **D29. Which of these activities could be carried out in the home setting in the future, so they have the highest degree of ease of implementation?** *(multiple answer)*

1. Functional reassessment of the patient
2. Physiatric interventions as infiltrative and symptomatic therapies
3. Programs for balance, sensory and cognitive disorders
4. Instrumental assessment of gait, balance, and posture
5. Rehabilitation activities without the use of rehabilitation machines
6. Rehabilitation activities using rehabilitation machines
7. Specialist consultation required (specify specialist )
8. Treatment and prevention of secondary complications
9. Assessment and management of neuropathic and nociceptive pain
10. Instrumental and clinical evaluation for use of tools such as baclofen pump or functional bandages
11. Respiratory re-education activities
12. Other (please specify )
13. Can't answer
14. None of these

### **D29.1 And which of these activities have the highest added value for this type of patient (patients with EDSS >6.5)?** (*multiple answer*)

1. Functional reassessment of the patient
2. Physiatric interventions as infiltrative and symptomatic therapies
3. Programs for balance, sensory and cognitive disorders
4. Instrumental assessment of gait, balance, and posture
5. Rehabilitation activities without the use of rehabilitation machines
6. Rehabilitation activities using rehabilitation machines
7. Request specialist consultation (specify specialist )
8. Treatment and prevention of secondary complications
9. Assessment and management of neuropathic and nociceptive pain
10. Instrumental and clinical evaluation for use of tools such as baclofen pump or functional bandages
11. Respiratory re-education activities
12. Other (please specify )
13. Can't answer
14. None of these

### **D30. In what cases is integrated home care (ADI) activated?** *(single answer)*

1. It is never activated
2. Enabled for most patients with EDSS >6.5
3. Activated mainly for (specify )
4. Can't answer

*If d30 code 2-3*

### **D31. What activities does the Integrated Home Care (ADI) perform at the patient's home?** *(multiple answer)*

1. Specialist visits
2. Physiatric examinations (for prescription of rehabilitation services)
3. Rehabilitation services (physiotherapy and speech therapy)
4. Nursing services
5. Health education/training of caregiver (family member, caregiver, volunteer)
6. Drawings, Hemogasanalysis
7. Electrocardiogram
8. Prescription of prescriptions
9. Other (specify: )

### **D32. What are the patient's care needs that Integrated Home Care (ADI) fails to cover?** *(multiple answer)*

1. Frequency of visits
2. Insufficient visit duration;
3. Educational support;
4. Psychological support
5. Motivational support
6. Other (specify: )

### **D33. How would an additional patient support service fill care needs not covered by ADI?** *(open answer)*

**D34. Finally, are you able to plan and carry out preventive educational activities or feedback of the educational interventions provided by the center at your center?**

| - preventive educational activities | □ Yes | □ No | □ I don't know. |
| --- | --- | --- | --- |
| - feedback of educational interventions | □ Yes | □ No | □ I don't know. |

**Supplementary 2**

Delphi method preliminary set of KPIs

| **Area** | **Type** | **Indicator** | **Numerator** | **Denominator** | **Threshold** | **Phase** | **Source** |
| --- | --- | --- | --- | --- | --- | --- | --- |
| Diagnostic examination | Process | **Time between first contact with a neurology facility and initiation of DMT therapy** | Number of patients newly diagnosed with MS in the year under review who started DMT within 90 days of first contact with the neurological structure in the same year | Part of the incident cohort that started a DMT from April 1 to December 31 | >80% | Diagnosis | Agenas / Gruppo di lavoro specializzato in neurologia (Inghilterra NHS) /NICE |
| Adequate follow-up frequency | Process | **Undergoing at least one neurological visit / patient / year** | Number of patients diagnosed with MS who have undergone at least one visit in the year under review | Total patients enrolled | >80% | Neurological follow-up | Agenas / PDTA Sardegna / Lazio / Gruppo di lavoro specializzato in neurologia (Inghilterra NHS) /NICE |
| Diagnostic examination | Process | **Performing at least one brain MRI per year for all patients on DMT therapy** | Number of patients diagnosed with MS on DMT therapy who had at least one brain MRI in the year under review | Number of MS patients on DMT therapy | >80% | Neurological follow-up | Agenas / PDTA Sardegna |
| Telemedicine | Process | **% Follow-up televisits** | Number of follow-up televisits | Total visits |  | Neurological follow-up |  |
| **Area** | **Type** | **Indicator** | **Numerator** | **Denominator** | **Threshold** | **Phase** | **Source** |
| Appropriate accesses  / Setting | Process | **% Inappropriate accesses to the ER (patients enrolled in the MS Center with accesses to the ER)** | Number of inappropriate accesses to the ER of MS patients enrolled in the MSC | Number of accesses to the ER of enrolled patients |  | Therapy | PDTA Emilia Romagna |
| Appropriate accesses  / Setting | Process | **% Patients using the Diagnostic PAC (Complex Clinical Package) (EDSS I and II)** | Number of MS PACs in the year, concluded within 30 days from the date the patient was newly diagnosed by a center | Number of diagnosed patients | It takes the previous year's value, if available, as a benchmark to evaluate the annual increase | Diagnosis | PDTA Lazio |
| Appropriate accesses  / Setting | Process | **Use of ordinary hospitalization for spinal taps** | Number of diagnosed with MS in the year during hospitalization, in which a spinal taping was performed as the main procedure, concluded within 30 days from the date indicated as enrolment of a newly diagnosed patient by a center | Number of patients diagnosed in the year with EDSS in the first two classes | 0 | Diagnosis | PDTA Lazio |
| Care Complexity | Volume | **% Patients enrolled with high care complexity** | % Patients with EDSS > 6.5 | Total patients enrolled |  | Therapy | PDTA Marche / Acta Medica Mediterranea dashboard of multiple sclerosis surveillance registry |
| **Area** | **Type** | **Indicator** | **Numerator** | **Denominator** | **Threshold** | **Phase** | **Source** |
| Care Complexity | Volume | **% MS patients in therapy at the MS center (calculated for I and II line)** | Patients on 1L therapy  Patients on 2L therapy | Total patients enrolled |  | Therapy | PDTA Abruzzo / Marche / Veneto / Emilia Romagna / Lazio/ Acta Medica Mediterranea dashboard of multiple sclerosis surveillance registry |
| Multidisciplinary approach | Process | **Level of multidisciplinary care enrolment** | Total scheduled services with other specialists | Total services performed with other specialists | >90% | Neurological follow-up | PDTA Marche |
| Patient Experience | Patient Experience | **QoL assessment through the MUSI QoL QUESTIONNAIRE** |  |  |  | Therapy | PDTA Calabria |
| Patient Experience | Patient Experience | **% Complaints received** | Number of complaints | Total patients enrolled | <1% | Therapy | PDTA Marche |
|  |  |  |  |  |  |  |  |
| **Area** | **Type** | **Indicator** | **Numerator** | **Denominator** | **Threshold** | **Phase** | **Source** |
| Rehabilitation pathway | Process | **% MS patients treated in inpatient rehabilitation services (in different settings) and territorial rehabilitation services** | Number of patients treated by rehabilitation services | Total patients enrolled |  | Rehabilitation | PDTA Emilia Romagna / Friuli Venezia Giulia / Lazio / Calabria |
| Rehabilitation pathway | Process | **Undergoing a physiatric examination in one year** | % Patients with at least 1 physiatric visit in 1 year | Total patients enrolled | 100% | Rehabilitation | PDTA Sardegna / Abruzzo |
| Rehabilitation pathway | Process | **Average number of contacts/year between neurologist and rehabilitation team per patient in therapy** | Average contacts between neurologist and rehabilitation team | Total patients enrolled |  | Rehabilitation | PDTA Friuli Venezia Giulia |
| Rehabilitation pathway | Process | **Level of integration: multidisciplinary rehabilitation management** | Deviations > 1 week between the time of assessment and the time prescribed for rehabilitation pathway |  | <10% | Rehabilitation | PDTA Marche |
| Diagnostic examination | Process | **% Brain MRI prescription** | Number of brain MRIs prescribed | Total patients enrolled |  | Neurological follow-up | PDTA Marche / Lazio / Sardegna |
|  |  |  |  |  |  |  |  |
| **Area** | **Type** | **Indicator** | **Numerator** | **Denominator** | **Threshold** | **Phase** | **Source** |
| Diagnostic examination | Process | **Average blood count tests / year per patient (calculated for first and second line)** | Number of blood counts for I line and II line patients | Total patients enrolled | >1 I line >2 II line | Neurological follow-up | PDTA Sardegna |
| Disease progression, complications, adverse events | Outcome | **% Patients with change in EDSS** | Patients with change in EDSS | Total patients enrolled |  | Therapy | PDTA Calabria |
| Disease progression, complications, adverse events | Outcome | **% Patients hospitalized for infectious complications** | Number of hospitalizations, non-rehabilitation, in the year under review with a primary or secondary discharge diagnosis of MS with one or more of the following diagnoses (pneumonia, urinary tract infection) | Total patients enrolled |  | Therapy | Agenas / PDTA Lazio / Veneto /Friuli Venezia Giulia / Lombardia |
| Disease progression, complications, adverse events | Outcome | **% Adverse events caused by drugs (DMTs)** | Number of adverse events caused by DMT | Number of patients on DMT therapy |  | Therapy | PDTA Emilia Romagna / Friuli Venezia Giulia / Calabria /Acta Medica Mediterranea dashboard of multiple sclerosis surveillance registry |
| **Area** | **Type** | **Indicator** | **Numerator** | **Denominator** | **Threshold** | **Phase** | **Source** |
| Patient centricity | Process | **% Integrated Care Plan (PAI) on the total of patients** | Number of MS patients with activated PAI | Total patients enrolled | Previous Year Reference | Cross | PDTA Calabria / Lazio / Emilia Romagna / Friuli Venezia Giulia |
| Patient centricity | Process | **Level of social and health integration** | Number of PAIs proposed by the MS Centre | Total number of PAIs performed for patients in the MS Center | 100% | Cross | PDTA Marche / Emilia Romagna / Friuli Venezia Giulia |
| Patient centricity | Process | **Average number of contacts/year between GP and neurologist per patient in therapy** | **Number of contacts** | Total patients enrolled | >10% | Cross | PDTA Friuli Venezia Giulia / Marche |
| Telemedicine | Process | **% Televisits for eligible patients** | Number of patients who have undergone at least 1 follow-up televisit in the year | Number of patients eligible for televisits |  | Neurological follow-up |  |
| Palliative care | Process | **% Advance Directives**^(*)^ | Advance Directives^(*)^ | Number of patients with EDSS > 7.5 |  |  |  |

(*) We referred to the tool provided by Italian Law 219/2017, called "Pianificazione condivisa delle cure" in Italian.

**Supplementary 3**

Maturity Model Self-Assessment Form. Each dimension has 4 levels, with the forth one being the characteristics of the optimal proximity care model.

Initial Diagnostic Suspicion Phase

Referral mechanisms:

Level 1. There is no structured referral mechanism from the territory to the MSC, so patients often go to the emergency room or are referred to other specialists. No training and/or awareness-raising activities are carried out for other specialists and GPs; absence of formalized and shared referral documents (e.g. Vademecum).

Level 2. There is no structured referral mechanism from the territory to the MSC, but clinicians use unstructured communication channels to facilitate referral. Training and/or awareness-raising activities have been carried out only for certain specialists within the MS Centre and the hospital; presence of formalized referral document, but not shared (e.g. Vademecum).

Level 3. There is a referral procedure from the territory to the MSC, formalized by the PDTA, but not well known by the territorial clinicians. Occasional training and awareness-raising actions only for some actors involved; presence of formalized and shared referral document (e.g. Vademecum).

Level 4. A referral procedure from the territory to the MS Centre is foreseen, formalized by the PDTA and well shared with all the territorial actors involved in the pathway. There are periodic awareness-raising and training actions for the actors involved in the pathway; the presence of a formalized and shared referral document (e.g. Vademecum) and the possibility of using Teleconsultation tools for suspected MS.

Coordination of actors involved:

Level 1. All communication between the actors involved in the MS patient's care process takes place when needed and in an unstructured way (e.g. by telephone, e-mail, personal contacts, etc.).

Level 2. The staff of the ward where the patient is being treated organize mono-disciplinary meetings in which aspects of the patient's care pathway are shared and discussed. Other professionals are possibly involved separately, outside the mono-disciplinary meetings.

Level 3. In mono-disciplinary meetings, other professionals who are asked to play an active role in the patient's care pathway are occasionally involved. Possible informal contacts with external professionals.

Level 4. There are codified multidisciplinary meetings, dedicated to the discussion of the patient's care pathway, which are regularly attended by experts from different disciplines and the case manager. Use of telemedicine tools for the involvement of territorial actors and/or other facilities.

Teleconsultation:

Level 1. No Teleconsultation initiative is implemented.

Level 2. Informal Teleconsultation initiatives are implemented: communication between professionals takes place through personal connections between clinicians.

Level 3. Use of non-institutionalized teleconsultation tools capable of tracking information, such as: e-mail, instant messaging tools, generalist video-conferencing systems, etc.

Level 4. Use of collaboration tools that make patient results and reports available, which can then be evaluated by the professionals involved at the same time.

Diagnosis and Therapy Phase

Collaboration with the territory:

Level 1. No collaboration with territorial actors is implemented.

Level 2. Partial division of tasks (e.g. examinations/visits) between actors at the Centre and territorial actors and contacts take place through personal connections between clinicians.

Level 3. Shared references for access to equipment and specialist visits, supported by a data exchange system in the catchment area and structuring of discussion/training meetings between all actors involved.

Level 4. Creation of a hospital-territory steering committee and formalization of a cross-setting pathway.

Management of service provision:

Level1. There are no dedicated slots for in-depth instrumental examinations (e.g. MRI) and there is no formalized territorial mapping of centers with suitable MRIs. Spinal tap not included in diagnostic protocols or in Day Hospital regimen.

Level 2. No dedicated slots for performing in-depth instrumental examinations; presence of informal list of centers with suitable MRIs. Spinal tap not included in diagnostic protocols or in Day Hospital regimen.

Level 3. Dedicated slots are available for in-depth instrumental examinations, with reservation done by the patient (e.g. MRI) and there is an informal list of centers with suitable MRIs. Spinal tap is provided by diagnostic protocols or in Day Hospital regimen.

Level 4. There are dedicated slots for in-depth instrumental examinations, the booking of which is managed by the Centre (e.g. MRI) and there is a territorial mapping of centers with suitable MRIs. Spinal tap is provided by diagnostic protocols or in Day Hospital regimen.

Teleconsultation:

Level 1. No Teleconsultation initiative is implemented.

Level 2. Informal Teleconsultation initiatives are implemented: communication between professionals takes place through personal connections between clinicians.

Level 3. Use of non-institutionalized teleconsultation tools capable of tracking information, such as: e-mail, instant messaging tools, generalist video-conferencing systems, etc.

Level 4. Use of collaboration tools that make patient results and reports available, which can then be evaluated by the professionals involved at the same time.

Care enrolment

Coordination of actors involved:

Level 1. All communication between the actors involved in the MS patient's care process takes place when needed and in an unstructured way (e.g. by telephone, e-mail, personal contacts, etc.).

Level 2. The staff of the ward where the patient is being treated organize mono-disciplinary meetings in which aspects of the patient's care pathway are shared and discussed. Other professionals are possibly involved separately, outside the mono-disciplinary meetings.

Level 3. In mono-disciplinary meetings, other professionals who are asked to play an active role in the patient's care pathway are occasionally involved. Possible informal contacts with external professionals.

Level 4. There are codified multidisciplinary meetings, dedicated to the discussion of the patient's care pathway, which are regularly attended by experts from different disciplines and the case manager. Use of telemedicine tools for the involvement of territorial actors and/or other facilities.

Provision of care enrolment:

Level 1. The specialist who first visits the patient defines diagnosis and treatment pathway. Absence of a rehabilitation care pathway.

Level 2. The specialist who first visits the patient defines the diagnosis and treatment pathway, occasionally involving other internal specialists. Presence of partial rehabilitation care pathway which is not formalized.

Level 3. The specialist who first visits the patient, according to company procedures, may request the support of the multidisciplinary team. Presence of rehabilitation care pathway, with difficulties in access.

Level 4. The multidisciplinary team defines the most suitable diagnosis and treatment pathway for the patient and the reference specialist, who takes charge of the patient, also involving territorial figures in the management of the pathway. Presence of structured rehabilitation care pathway and activation of the individual rehabilitation plan.

Case management:

Level 1. There is no optimization of patient access and no case manager or figure in charge of managing the pathway. Absence of direct communication channels with the MS Centre.

Level 2. The patient accesses the MSC numerous times (to pick-up the treatment plan, pick-up oral/inj therapies, visits and check-ups...) and occasionally the Centre manages to match two services; although there is no case manager or figure in charge of managing the pathway. The patient, in case of need, refers to the neurologist’s personal email.

Level 3. Case management exists but is performed by sub-optimal staff who is inadequately trained or engaged in too many activities; sharing of the treatment plan by email or at the time of the visit; collection of oral and subcutaneous therapies on the territory. There is a dedicated telephone number for the MSC, but insufficient staff to handle calls.

Level 4. Case management exists, appropriately trained and dedicated to the management of MS patients' care including aspects of physical, social, psychological, and existential needs; sharing of the treatment plan by email or at the time of the visit, which can also be carried out on remotely through Televisits; collection of oral and subcutaneous therapies on the territory or via home delivery services. There is a dedicated number, dedicated email box and dedicated staff to manage issues.

Clinical follow-up

Management of service provision:

Level1. There are no dedicated slots for in-depth instrumental examinations (e.g. MRI) and there is no formalized territorial mapping of centers with suitable MRIs. Spinal tap not included in diagnostic protocols or in Day Hospital regimen.

Level 2. No dedicated slots for performing in-depth instrumental examinations; presence of informal list of centers with suitable MRIs. Spinal tap not included in diagnostic protocols or in Day Hospital regimen.

Level 3. Dedicated slots are available for in-depth instrumental examinations, with reservation done by the patient (e.g. MRI) and there is an informal list of centers with suitable MRIs. Spinal tap is provided by diagnostic protocols or in Day Hospital regimen.

Level 4. There are dedicated slots for in-depth instrumental examinations, the booking of which is managed by the Centre (e.g. MRI) and there is a territorial mapping of centers with suitable MRIs. Spinal tap is provided by diagnostic protocols or in Day Hospital regimen.

Teleconsultation:

Level 1. No Teleconsultation initiative is implemented.

Level 2. Informal Teleconsultation initiatives are implemented: communication between professionals takes place through personal connections between clinicians.

Level 3. Use of non-institutionalized teleconsultation tools capable of tracking information, such as: e-mail, instant messaging tools, generalist video-conferencing systems, etc.

Level 4. Use of collaboration tools that make patient results and reports available, which can then be evaluated by the professionals involved at the same time.

Televisit:

Level 1. No Televisit initiative implemented: the patient is managed in presence and/or by telephone without the support of digital systems.

Level 2. Use of personal tools (voice calls from private phones) and instant messaging (whatsapp) for remote patient management.

Level 3. Use of Televisit tools that allow real time examination of patients (teams, zoom...), not institutionalized by the organization.

Level 4. Use of formalized Televisit tools capable of remote specialist examinations and for the treatment plan definition.

Rehabilitation follow-up

Rehabilitation pathway:

Level 1. Absence of internal pathway dedicated to rehabilitation care and absence of territorial mapping of appropriate rehabilitation centers.

Level 2. Possibility of physiatrist consultation within the hospital, by means of a referral and booking by the patient; but absence of territorial mapping of appropriate rehabilitation centers.

Level 3. Internal pathway dedicated to rehabilitation care, through agreements with rehabilitation medicine and presence of informal list of territorial appropriate rehabilitation centers. Sporadic communication between the MSC and the rehabilitation facility, based on personal connections between clinicians.

Level 4. Internal pathway dedicated to rehabilitation care, with a physiatrist dedicated to the MSC, who activates the individual rehabilitation plan and presence of territorial mapping of appropriate rehabilitation centers, with agreements for taking charge. Presence of structured and periodic touchpoints between neurologist and physiatrist/physiotherapist, also through telemedicine tools (telerehabilitation).

Teleconsultation:

Level 1. No Teleconsultation initiative is implemented.

Level 2. Informal Teleconsultation initiatives are implemented: communication between professionals takes place through personal connections between clinicians.

Level 3. Use of non-institutionalized teleconsultation tools capable of tracking information, such as: e-mail, instant messaging tools, generalist video-conferencing systems, etc.

Level 4. Use of collaboration tools that make patient results and reports available, which can then be evaluated by the professionals involved at the same time.

Telerehabilitation:

Level 1. No Telerehabilitation initiative is implemented: the patient is managed in presence.

Level 2. Use of unstructured platforms for remote physiatrists check-ups, but not for rehabilitation activities.

Level 3. Physiatrists and rehabilitators use personal instant messaging tools or generalist software to contact patients and perform partial remote support during rehabilitation activities.

Level 4. Using Telerehabilitation: carrying out a complete rehabilitation session remotely.

Cross pathway

Availability of clinical support systems:

Level 1. Completely paper-based clinical records and forms: only some specific clinical documents are managed digitally (e.g. reports).

Level 2. Presence of the organization’s software with computerized medical records, where the basic data of the patient's care pathway and computerized management of visits are stored. Presence of computerized schedules by means of files saved on the computer (e.g. word sheet, excel...).

Level 3. Presence of the organization’s software with computerized medical records with advanced functionalities (e.g. detection of vital parameters from monitoring devices). Presence of computerized diaries using general tools (e.g. google calendar...).

Level 4. Presence of the organization’s software with computerized medical records with advanced functions (e.g. detection of vital parameters from monitoring devices) and medical records usable on mobile devices. Presence of computerized diaries created ad hoc for the pathology.

Collaboration tools:

Level 1. Communication between the actors involved in the patient care pathway takes place in an unstructured way (e.g. telephone, email, Skype, ...). Examination reports exclusively on paper.

Level 2. Communication between the actors involved in the patient care pathway takes place in an unstructured manner, tracked by a digital system in order to keep track of all interactions affecting the care pathway during multidisciplinary meetings. Reports shared digitally and viewable by MSC staff.

Level 3. Communications between the actors involved in the patient care pathway take place in a structured manner and are tracked with a digital system in order to keep track of all interactions affecting the care pathway during multidisciplinary meetings. External assessments shared digitally; internal assessments viewable from the organization’s platform.

Level 4. Presence of decision-making workflow with communication between actors through digitization and presence of shared agendas between specialists for organizing multidisciplinary visits. Referrals digitally stored in patients' medical records, which can be consulted by all professionals involved.

KPI monitoring:

Level 1. The clinical center does not monitor indicators.

Level 2. The clinical center monitors clinically defined and specifically developed indicators for monitoring the MS patient's care pathway.

Level 3. The clinical center monitors clinical and economic indicators specifically developed for monitoring the MS patient's care pathway.

Level 4. The clinical centre monitors clinical, economic and management indicators (time and quality), specifically developed for monitoring the MS patient's care pathway. The connection with the territory and telemedicine are part of the analyzed indicators.

ICP and dedicated multidisciplinary team:

Level 1. There is no specific PDTA for MS. Within the clinical center there are no figures specifically designated for the management of patients with Multiple Sclerosis.

Level 2. There is a specific PDTA for MS, which is, however, obsolete as it was formalized before 2016. Within the clinical center, a number of figures have been defined specifically for the management of MS patients. Absence of dedicated pathways or formalized contacts with other specialties, but recourse to unstructured modes of interaction.

Level 3. There is a specific regional PDTA, on average obsolete as it was formalized between 2016 and 2019 and implemented by the company. There is an multidisciplinary team, formalized within the company's PDTA, in which the specialists who act as contact persons for the management of patients are indicated (e.g. neurologist, physiatrist...) and periodic structured multidisciplinary meetings are scheduled.

Level 4. There is a specific regional PDTA, updated according to GLASM indications and implemented by the company. There is a network of formalized relations with territorial actors, involved through telemedicine. The possible presence of a case manager, with both an administrative and management role, would facilitate multidisciplinarity.

**Supplementary 4**

Maturity Model preliminary pre-post analysis results. For each MSC the level of every dimension is reported for the 2 periods as well as the change that has occurred. Additionally, for each Center the mean level of dimensions for T0 and T1 are reported as well and the mean difference between the periods. The dimensions are the following: 1. Referral mechanisms 2. Coordination of actors involved 3. Teleconsultation 4. Collaboration with the territory 5. Management of service provision 6. Teleconsultation 7. Coordination of actors involved 8. Provision of care enrolment 9. Case management 10. Management of service provision 11. Teleconsultation 12. Televisit 13. Rehabilitation pathway 14. Teleconsultation 15. Telerehabilitation 16. Availability of clinical support systems 17. Collaboration tools 18. KPI monitoring 19. ICP and dedicated multidisciplinary team.

| **MSC** | | **Period** | **Maturity Model Dimensions** | | | | | | | | | | | | | | | | | | | **Mean** |
| --- | --- | --- | --- | --- | --- | --- | --- | --- | --- | --- | --- | --- | --- | --- | --- | --- | --- | --- | --- | --- | --- | --- |
|  |  |  | **1** | **2** | **3** | **4** | **5** | **6** | **7** | **8** | **9** | **10** | **11** | **12** | **13** | **14** | **15** | **16** | **17** | **18** | **19** |  |
| **A** | T0 | | 1 | 2 | 1 | 1 | 1 | 1 | 1 | 2 | 3 | 1 | 1 | 1 | 2 | 1 | 1 | 1 | 1 | 1 | 3 | 1,4 |
|  | T1 | | 2 | 2 | 3 | 1 | 4 | 3 | 2 | 1 | 4 | 4 | 3 | 3 | 2 | 3 | 1 | 2 | 2 | 1 | 2 | 2,4 |
|  | Δ | | 1 | 0 | 2 | 0 | 3 | 2 | 1 | -1 | 1 | 3 | 2 | 2 | 0 | 2 | 0 | 1 | 1 | 0 | -1 | 1,0 |
| **B** | T0 | | 1 | 2 | 1 | 2 | 2 | 1 | 1 | 2 | 2 | 2 | 1 | 1 | 2 | 1 | 1 | 3 | 2 | 1 | 2 | 1,6 |
|  | T1 | | 1 | 1 | 1 | 1 | 4 | 3 | 1 | 1 | 2 | 4 | 3 | 2 | 1 | 1 | 1 | 1 | 1 | 1 | 2 | 2,0 |
|  | Δ | | 0 | -1 | 0 | -1 | 2 | 2 | 0 | -1 | 0 | 2 | 2 | 1 | -1 | 0 | 0 | -2 | -1 | 0 | 0 | 0,1 |
| **C** | T0 | | 1 | 3 | 2 | 1 | 1 | 2 | 2 | 2 | 2 | 1 | 2 | 1 | 3 | 2 | 1 | 1 | 1 | 1 | 2 | 1,6 |
|  | T1 | | 3 | 1 | 1 | 2 | 3 | 1 | 1 | 4 | 3 | 4 | 1 | 1 | 2 | 1 | 1 | 1 | 1 | 3 | 4 | 2,0 |
|  | Δ | | 2 | -2 | -1 | 1 | 2 | -1 | -1 | 2 | 1 | 3 | -1 | 0 | -1 | -1 | 0 | 0 | 0 | 2 | 2 | 0,4 |
| **D** | T0 | | 1 | 2 | 2 | 1 | 3 | 2 | 2 | 3 | 2 | 3 | 2 | 2 | 2 | 2 | 1 | 2 | 2 | 1 | 2 | 1,9 |
|  | T1 | | 3 | 4 | 4 | 4 | 3 | 4 | 4 | 3 | 4 | 3 | 4 | 4 | 3 | 3 | 2 | 3 | 4 | 4 | 4 | 3,5 |
|  | Δ | | 2 | 2 | 2 | 3 | 0 | 2 | 2 | 0 | 2 | 0 | 2 | 2 | 1 | 1 | 1 | 1 | 2 | 3 | 2 | 1,6 |
| **E** | T0 | | 1 | 1 | 1 | 1 | 2 | 1 | 2 | 3 | 2 | 2 | 1 | 2 | 3 | 1 | 1 | 2 | 1 | 1 | 2 | 1,6 |
|  | T1 | | 3 | 3 | 3 | 4 | 4 | 3 | 3 | 4 | 3 | 4 | 3 | 1 | 4 | 3 | 1 | 2 | 2 | 2 | 4 | 2,9 |
|  | Δ | | 2 | 2 | 2 | 3 | 2 | 2 | 1 | 1 | 1 | 2 | 2 | -1 | 1 | 2 | 0 | 0 | 1 | 1 | 2 | 1,4 |
| **F** | T0 | | 1 | 1 | 2 | 2 | 1 | 1 | 1 | 2 | 3 | 1 | 1 | 2 | 2 | 1 | 1 | 1 | 1 | 1 | 1 | 1,4 |
|  | T1 | | 1 | 2 | 1 | 2 | 4 | 3 | 2 | 4 | 4 | 4 | 3 | 2 | 4 | 2 | 1 | 2 | 2 | 3 | 1 | 2,5 |
|  | Δ | | 0 | 1 | -1 | 0 | 3 | 2 | 1 | 2 | 1 | 3 | 2 | 0 | 2 | 1 | 0 | 1 | 1 | 2 | 0 | 1,1 |
| **G** | T0 | | 2 | 1 | 1 | 1 | 2 | 1 | 2 | 1 | 1 | 2 | 1 | 1 | 1 | 1 | 1 | 1 | 1 | 1 | 3 | 1,3 |
|  | T1 | | 2 | 3 | 3 | 1 | 4 | 3 | 1 | 1 | 3 | 4 | 1 | 2 | 1 | 1 | 1 | 1 | 1 | 1 | 4 | 2,0 |
|  | Δ | | 0 | 2 | 2 | 0 | 2 | 2 | -1 | 0 | 2 | 2 | 0 | 1 | 0 | 0 | 0 | 0 | 0 | 0 | 1 | 0,7 |
| **H** | T0 | | 1 | 2 | 2 | 2 | 1 | 2 | 3 | 3 | 3 | 1 | 2 | 2 | 3 | 2 | 2 | 3 | 1 | 1 | 3 | 2,1 |
|  | T1 | | 2 | 2 | 2 | 2 | 4 | 2 | 3 | 3 | 3 | 4 | 2 | 2 | 3 | 2 | 2 | 4 | 2 | 3 | 3 | 2,6 |
|  | Δ | | 1 | 2 | 2 | 2 | 3 | 0 | 0 | 0 | 0 | 3 | 0 | 0 | 0 | 0 | 0 | 1 | 1 | 2 | 0 | 0,6 |
| **I** | T0 | | 1 | 1 | 1 | 2 | 4 | 2 | 1 | 2 | 4 | 4 | 2 | 2 | 2 | 1 | 1 | 2 | 1 | 1 | 1 | 1,8 |
|  | T1 | | 1 | 2 | 1 | 3 | 4 | 1 | 4 | 3 | 4 | 4 | 1 | 1 | 1 | 1 | 1 | 2 | 2 | 1 | 2 | 2,1 |
|  | Δ | | 0 | 1 | 0 | 1 | 0 | -1 | 3 | 1 | 0 | 0 | -1 | -1 | -1 | 0 | 0 | 0 | 1 | 0 | 1 | 0,2 |
| **J** | T0 | | 1 | 1 | 2 | 2 | 1 | 1 | 1 | 2 | 3 | 1 | 1 | 2 | 2 | 1 | 1 | 1 | 1 | 1 | 1 | 1,4 |
|  | T1 | | 2 | 1 | 3 | 2 | 4 | 2 | 4 | 4 | 4 | 4 | 2 | 1 | 4 | 2 | 1 | 2 | 2 | 1 | 3 | 2,5 |
|  | Δ | | 1 | 0 | 1 | 0 | 3 | 1 | 3 | 2 | 1 | 3 | 1 | -1 | 2 | 1 | 0 | 1 | 1 | 0 | 2 | 1,2 |
| **K** | T0 | | 1 | 1 | 1 | 2 | 2 | 1 | 2 | 2 | 2 | 2 | 1 | 3 | 2 | 1 | 1 | 1 | 2 | 1 | 2 | 1,6 |
|  | T1 | | 2 | 1 | 1 | 1 | 4 | 1 | 1 | 2 | 2 | 4 | 1 | 2 | 2 | 1 | 1 | 2 | 1 | 1 | 3 | 1,7 |
|  | Δ | | 1 | 0 | 0 | -1 | 2 | 0 | -1 | 0 | 0 | 2 | 0 | -1 | 0 | 0 | 0 | 1 | -1 | 0 | 1 | 0,2 |

The data considers 11 MSCs. The dimensions are rated from 1 to 4, with 1 being lowest and 4 highest. The average Δ is measured by calculating the difference between the 2 periods. Period T0 refers to the baseline measure, while T1 is calculated after at least 6 months from the first measurement.

**Supplementary 5**

Delphi method: KPI inclusion

| **KPI** | **Round 1** | **Round 2** |
| --- | --- | --- |
| % Inappropriate accesses to the ER (patients enrolled in the MS Center with accesses to the ER) | Uncertain | Included |
| % Patients using the Diagnostic PAC (Complex Clinical Package) (EDSS I and II) | Uncertain | Excluded |
| Use of ordinary hospitalization for spinal taps | Uncertain | Excluded |
| % Patients enrolled with high care complexity | Uncertain | Included |
| % MS patients in therapy at the MS center (calculated for I and II line) | Included |  |
| Undergoing at least one neurological visit / patient / year | Included |  |
| Level of multidisciplinary care enrolment | Uncertain | Included |
| QoL assessment through the MUSI QoL Questionnaire | Uncertain | Excluded |
| % Complaints received | Uncertain | Excluded |
| % MS patients treated in inpatient rehabilitation services (in different settings) and territorial rehabilitation services | Uncertain | Included |
| Undergoing a physiatric examination in one year | Uncertain | Excluded |
| Average number of contacts/year between neurologist and rehabilitation team per patient in therapy | Uncertain | Excluded |
| Level of integration: multidisciplinary rehabilitation management | Uncertain | Excluded |
| Performing at least one brain MRI per year for all patients on DMT therapy | Included |  |
| % Brain MRI prescription | Uncertain | Excluded |
| Average blood count tests / year per patient (calculated for first and second line) | Uncertain | Excluded |
| Time between first contact with a neurology facility and initiation of DMT therapy | Included |  |
| % Patients with change in EDSS | Uncertain | Excluded |
| % Patients hospitalized for infectious complications | Uncertain | Included |
| % Adverse events caused by drugs (DMTs) | Uncertain | Excluded |
| % Integrated Care Plan (PAI) on the total of patients | Uncertain | Excluded |
| Level of social and health integration | Uncertain | Excluded |
| Average number of contacts/year between GP and neurologist per patient in therapy | Uncertain | Excluded |
| % Follow-up televisits | Uncertain | Excluded |
| % Televisits for eligible patients | Uncertain | Excluded |
| % Advance Directives^(*)^ | Uncertain | Excluded |

(*) We referred to the tool provided by Italian Law 219/2017, called "Pianificazione condivisa delle cure" in Italian.

| **Dimensions** | **Round 1** | **Round 2** |
| --- | --- | --- |
| Referral mechanisms | Uncertain | Excluded |
| Coordination of actors involved | Included |  |
| Teleconsultation | Included |  |
| Collaboration with the territory | Uncertain | Included |
| Management of service provision | Included |  |
| Teleconsultation | Included |  |
| Coordination of actors involved | Included |  |
| Provision of care enrolment | Included |  |
| Case management | Included |  |
| Management of service provision | Included |  |
| Teleconsultation | Included |  |
| Televisita | Uncertain | Included |
| Rehabilitation pathway | Included |  |
| Teleconsultation | Included |  |
| Telerehabilitation | Uncertain | Excluded |
| Availability of clinical support systems | Included |  |
| Collaboration tools | Uncertain | Excluded |
| KPI monitoring | Uncertain | Included |
| ICP and dedicated multidisciplinary team | Included |  |
| Palliative care | Uncertain | Included |

**Supplementary 6**

Delphi method: Maturity Model inclusion
